# Selection and validation of reference gene for RT-qPCR studies in co-culture system of mouse cementoblasts and periodontal ligament cells

**DOI:** 10.1186/s13104-022-05948-x

**Published:** 2022-02-15

**Authors:** Jiawen Yong, Sabine Groeger, Gisela Ruiz-Heiland, Sabine Ruf

**Affiliations:** 1 Department of Orthodontics, Faculty of Medicine, Justus Liebig University of Giessen, Schlangenzahl 14, 35392 Giessen, Germany; 2 Department of Periodontics, Faculty of Medicine, Justus Liebig University of Giessen, Giessen, Germany

**Keywords:** Reference genes, Cementoblasts, Periodontal ligament cells, RT-qPCR, Co-culture

## Abstract

**Objective:**

RT-qPCR is a reliable method for gene expression analysis, but the accuracy of the quantitative data depends on the appropriate selection of reference genes. A Co-culture system consisting of periodontal ligament cells (SV-PDL) and cementoblasts (OCCM-30) to investigate the crosstalk between these two cell lines under orthodontic condition is essential for experimental orthodontic setups in-vitro. Therefore, we aimed to identify a set of reliable reference genes suitable for RT-qPCR studies for prospective co-culture systems of OCCM-30 and SV-PDL cells.

**Results:**

The results demonstrated that *PPIB*, *GUSB* and *RPLP0* turned out to be the three most stable reference genes for OCCM-30 in the co-culture system, while *PPIB*, *POLR2A* and *RPLP0* have the three highest rankings for SV-PDL cells in the co-culture system. The most stable gene combination were *PPIB* and *POLR2A* in the co-culture system. In conclusion, *PPIB* is overall the most stably expressed reference gene for OCCM-30 or SV-PDL cell line in the system. The combination of *PPIB* and *POLR2A* as reference genes are indicated to be the potential and mandatory to obtain accurate quantification results for normalizing RT-qPCR data in genes of interest expression in these two cell lines co-culture systems.

**Supplementary Information:**

The online version contains supplementary material available at 10.1186/s13104-022-05948-x.

## Introduction

Cementoblasts are located on the cementum covered root surface and have the lifelong capability to produce cementum [1]. Periodontal ligament cells are fibroblast-like cells characterized by collagen production [2].

Reverse Transcriptase-quantitative polymerase chain reaction (RT-qPCR) [3] is a versatile molecular technique for quantification of the expression of genes of interest due to the method’s merits concerning its high sensitivity, simplicity and specificity as well as its accuracy [4, 5]. Reference genes are considered to be consistently expressed in various tissues and treatments [6], which guarantee precise gene expression quantification by accurate and valid data normalization [7].

Our laboratory will deliver a co-culture system of periodontal ligament cells with cementoblasts which in-vitro is used to mimic the biological conditions to explore the interaction between these two-cell lines [8–11]. Therefore, a commonly stable reference gene selection is of vital role for the co-culture system RT-qPCR experiment set.

Together from previous studies [12–15], the ribosomal 60S protein L22 (*RPL22*), Peptidylprolyl isomerase B (*PPIB*), polymerase RNA II polypeptide A (*POLR2A*), Ribosomal protein, large, P0 (*RPLP0*), glucuronidase, beta (*GUSB*), Actin-beta (*β-actin*), TATA-binding protein (*TBP*), ubiquitin C (*UBC*), glyceraldehyde-3-phosphate dehydrogenase (*GAPDH*), Tyrosine 3-monooxygenase/tryptophan 5-mono-oxygenase activate protein, zeta (*YWHAZ*), eukaryotic translation elongation factor 1 alpha 1 (*EEF1A1*), Ribosomal protein L18 (*RPL*) and beta-5 class I (*TUBB*) were identified as reliable reference genes.

Since co-culture system could be a promising method to analyze the biological effect response to mimic orthodontically induced tooth movement in-vitro. The identification of the most reliable reference genes for RT-qPCR analysis on OCCM-30 and SV-PDL, is an essential step to facilitate further research in this area.

## Main text

### Materials and methods

#### Mono cell culture

Immortalized murine mouse cementoblast (OCCM-30) cell line [16] and immortalized mouse murine periodontal ligament (SV-PDL) cells [17] were provided by Prof. Martha J. Somerman (Laboratory of Oral Connective Tissue Biology, NIH, Bethesda, USA). The optimal density of OCCM-30 [18] and SV-PDL cells [17] were previously determined, thus 60—100% confluence status of cells were used for cell confluence experiments [15].

Both cells were cultured at a density of 1*10^6^ cell/well in D-MEM (31885-023, Gibco) containing 10% FBS (10270-106, Gibco), 1% Penicillin/Streptomycin (15140-122, Gibco) and 1% HEPES (15630-056, Gibco) in a humidified atmosphere of 5% CO_2_ at 37 °C.

#### Direct cell–cell contact culture

The direct cell–cell contact system was established by seeding each cell line by same number of 1*10^6^ cells/well in the same 6-well plate. After incubation overnight to allow for firm adherence to the bottom, the cell–cell contact system was established [19]. For the experiment, control group (as 0 h) was set when cells reached approximately 60% confluence. The mRNA was harvested at 0, 12 and 24 h at the same day.

#### Co-culture system

The OCCM-30/SV-PDL co-culture system was established through 6-well plate and ThinCert^@^ Cell Culture Inserts (pore size 0.4 µm, porosity/transparent membrane) (657,641, Greiner Bio-One) enabling the cells to exchange soluble factors [8] as previous described [11]. Briefly, the SV-PDL cells (1*10^6^ cell/well) were seeded into 6-well plate and OCCM-30 cells (1*10^6^ cell/well) were seeded into the ThinCert^@^ inserts. After 6 h cultivation to allow for adherence, the inserts containing OCCM-30 cells are placed on top in the 6-well plate containing SV-PDL cells (Fig. 2A) on bottom. Then, the co-culture system was established after co-incubation for another 10 h. For the experiment, control group (as 0 h) was set when cells reached approximately 60% confluence. The mRNA was harvested at 0, 12, 24 h at the same day.

#### RT-qPCR analysis

Cells were harvested with 350 μL buffer RLT (Qiagen, Germany). Afterwards, RNA was isolated with RNase Mini Kit (Qiagen, Germany) following an on-column DNA digestion (RNase-Free DNase, Qiagen, Germany) including DNase step for removal of genomic DNA. After isolation, the eluted RNA purity and quantity of each sample was verified photometrically by OD readings of the A260/280 nm ratio (Nanodrop 2000, Thermo Fisher Scientific, USA).

For every RT-qPCR 20 μL volume reaction, we used 8 μL DNase-free water (Sigma-Aldrich), 10 μL SsoAdvanced™ Universal SYBR Green Supermix (Bio-Rad), 1.0 μL cDNA and 1.0 μL primer [20]. Primers are designed by Bio-Rad (Additional file 1: Table S1). For analysis, a C_q_ cut-off of 40 was applied.

**Table 1:** The rank of candidate reference gene stability for monocultured OCCM-30 or SV-PDL, direct cell–cell culture, co-cultured OCCM-30 cells and co-cultured SV-PDL cells, overall ranks were calculated by the four algorithms application (geNorm, NormFinder, comparative ΔC_q_ and BestKeeper, respectively)

Rank	Methods			geNorm			NormFinder			Comparative ΔC_q_			BestKeeper			
	Ranking order	Ranking sum		Ranking order	Stability value (M)		Ranking order	Stability value		Ranking order	Stability value		Ranking order	Stability value (r)	SD (± C_q_)	CV (% C_q_)
Monocultured cementoblasts (OCCM-30)																
(1)	**RPL22**	13		**GUSB**	0.032		**TBP**	0.065		**RPL22**	0.22		**PPIB**	0.750	0.470	2.278
(2)	**PPIB**	17		**POLR2A**	0.037		**PPIB**	0.065		**RPLP0**	0.26		**YWHAZ**	0.685	1.541	7.321
(3)	**POLR2A**	21		**RPL22**	0.042		**β-actin**	0.092		**POLR2A**	0.40		**GAPDH**	0.679	0.390	2.282
(4)	RPLP0	23		RPLP0	0.058		RPL22	0.136		GUSB	0.40		TBP	0.620	0.503	2.121
(5)	GUSB	23		UBC	0.063		RPL	0.212		UBC	0.53		RPL22	0.612	0.662	2.679
(6)	β-actin	24		β-actin	0.072		RPLP0	0.538		PPIB	0.54		β-actin	0.453	0.543	3.208
(7)	TBP	24		GAPDH	0.083		POLR2A	0.719		EEF1A1	0.59		RPL	0.172	0.204	0.790
(8)	UBC	27		PPIB	0.090		GUSB	0.831		TBP	0.59		UBC	-0.038	0.489	1.756
(9)	GAPDH	32		YWHAZ	0.095		UBC	0.859		β-actin	0.64		POLR2A	-0.115	0.197	0.875
(10)	YWHAZ	35		EEF1A1	0.113		EEF1A1	1.005		RPL	0.81		GUSB	-0.282	0.331	1.393
(11)	EEF1A1	39		TBP	0.183		GAPDH	2.421		GAPDH	2.12		RPLP0	-0.428	0.152	0.988
(12)	RPL	-					YWHAZ	2.461		YWHAZ	2.29		EEF1A1	-0.683	0.472	2.774
(13)	TUBB	-								TUBB	13.85		TUBB	-0.986	0.295	0.795
Monocultured periodontal ligament cells (SV-PDL)																
(1)	**GUSB**	14		**GUSB**	0.003		**EEF1A1**	0.112		**GUSB**	0.18		**TBP**	0.928	0.447	1.766
(2)	**RPLP0**	19		**GAPDH**	0.004		**GUSB**	0.112		**RPLP0**	0.19		**RPL22**	0.921	0.762	2.951
(3)	**RPL22**	22		**RPLP0**	0.007		**POLR2A**	0.123		**EEF1A1**	0.19		**UBC**	0.916	0.660	2.360
(4)	TBP	22		β-actin	0.021		β-actin	0.124		POLR2A	0.24		YWHAZ	0.910	0.455	2.103
(5)	β-actin	22		PPIB	0.031		TBP	0.124		RPL22	0.26		β-actin	0.905	0.537	3.093
(6)	PPIB	26		RPL22	0.040		RPLP0	0.125		TUBB	0.31		GAPDH	0.868	0.518	2.894
(7)	POLR2A	27		UBC	0.045		PPIB	0.206		PPIB	0.51		PPIB	0.739	0.446	2.094
(8)	GAPDH	30		TBP	0.050		TUBB	0.282		TBP	0.53		RPLP0	0.440	0.126	0.818
(9)	TUBB	36		POLR2A	0.075		RPL22	0.475		β-actin	0.66		RPL	0.355	0.250	0.996
(10)	EEF1A1	38		TUBB	0.100		RPL	0.526		RPL	0.92		GUSB	0.328	0.138	0.561
(11)	YWHAZ	49		EEF1A1	0.127		GAPDH	5.241		GAPDH	4.98		POLR2A	0.169	0.226	0.989
(12)	UBC	-		YWHAZ	0.850		YWHAZ	6.351		YWHAZ	5.99		TUBB	0.125	0.268	0.930
(13)	RPL	-								UBC	10.46		EEF1A1	-0.292	0.168	0.967
Direct cell–cell contact cultured of OCCM-30 and SV-PDL																
(1)	**GUSB**	7		**POLR2A**	0.086		**GUSB**	0.005		**RPLP0**	0.29		**POLR2A**	0.912	0.57	2.27
(2)	**POLR2A**	8		**GUSB**	0.089		**POLR2A**	0.007		**GUSB**	0.48		**GUSB**	0.889	0.62	2.36
(3)	**RPLP0**	14		**RPL22**	0.11		**RPL22**	0.019		**PPIB**	0.48		**PPIB**	0.872	1.07	4.83
(4)	RPL22	15		RPLP0	0.212		RPLP0	0.029		POLR2A	0.59		RPL22	0.784	0.62	2.22
(5)	PPIB	16		PPIB	0.399		PPIB	0.058		RPL22	1.09		RPLP0	0.575	0.31	2.00
Co-cultured OCCM-30 control																
(1)	**PPIB**	8		**PPIB**	0.211		**GUSB**	0.006		**RPLP0**	0.31		**RPL22**	0.986	1.5	5.31
(2)	**GUSB**	10		**POLR2A**	0.224		**PPIB**	0.013		**GUSB**	1.00		**PPIB**	0.977	0.95	4.34
(3)	**RPLP0**	14		**RPLP0**	0.325		**RPL22**	0.028		**PPIB**	1.02		**GUSB**	0.977	0.94	3.46
(4)	POLR2A	14		GUSB	0.347		POLR2A	0.040		POLR2A	1.03		POLR2A	0.658	1.01	3.92
(5)	RPL22	14		RPL22	0.704		RPLP0	0.045		RPL22	1.59		RPLP0	0.220	0.26	1.65
Co-cultured OCCM-30 with 3 indicated time (n = 18)																
(1)	**PPIB**	9		**RPLP0**	0.409		**PPIB**	0.016		**RPLP0**	0.63		**PPIB**	0.949	0.72	3.25
(2)	**GUSB**	9		**GUSB**	0.478		**GUSB**	0.021		**POLR2A**	0.74		**GUSB**	0.767	0.6	2.18
(3)	**RPLP0**	11		**PPIB**	0.515		**POLR2A**	0.027		**GUSB**	0.78		**RPL22**	0.712	0.98	3.54
(4)	POLR2A	14		POLR2A	0.565		RPL22	0.031		PPIB	0.78		RPLP0	0.500	0.49	2.9
(5)	RPL22	17		RPL22	0.840		RPLP0	0.035		RPL22	1.27		POLR2A	0.427	0.64	2.48
Co-cultured SV-PDL control																
(1)	**PPIB**	9		**RPLP0**	0.308		**PPIB**	0.004		**RPLP0**	0.11		**POLR2A**	0.998	0.65	2.59
(2)	**POLR2A**	11		**PPIB**	0.309		**RPL22**	0.004		**PPIB**	0.52		**RPL22**	0.983	0.68	2.59
(3)	**RPLP0**	12		**POLR2A**	0.378		**POLR2A**	0.009		**RPL22**	0.62		**GUSB**	0.980	0.53	1.85
(4)	RPL22	12		GUSB	0.390		GUSB	0.011		POLR2A	0.74		PPIB	0.973	0.47	2.17
(5)	GUSB	16		RPL22	0.732		RPLP0	0.023		GUSB	0.78		RPLP0	0.700	0.09	0.61
Co-cultured SV-PDL with 3 indicated time (n = 18)																
(1)	**PPIB**	8		**POLR2A**	0.161		**PPIB**	0.005		**RPLP0**	0.22		**PPIB**	0.980	0.31	1.44
(2)	**POLR2A**	10		**GUSB**	0.165		**RPLP0**	0.015		**PPIB**	0.42		**POLR2A**	0.823	0.5	1.98
(3)	**RPLP0**	11		**RPLP0**	0.214		**GUSB**	0.016		**POLR2A**	0.60		**GUSB**	0.774	0.54	2.04
(4)	GUSB	12		PPIB	0.218		POLR2A	0.017		GUSB	0.60		RPL22	0.746	0.57	2.02
(5)	RPL22	19		RPL22	0.422		RPL22	0.019		RPL22	0.74		RPLP0	0.646	0.19	1.21

A higher rank denotes lower expression stability. Individual primer efficiency was taken in to account by the abbreviations of C_q_ (threshold cycle), SD (standard deviation), CV (coefficient of variation) and r (Pearson’s correlation coefficient). The genes are ordered form the highest to the lowest ranking

#### Stability assessment and statistical analysis

Statistical analysis regarding reference gens stability was performed by using four different mathematical procedures: geNorm (qBase + , Biogazelle) [21], NormFinder (version 0.953) [22], BestKeeper (version 1) [23] and Comparative ΔC_q_ method [24]. The values of cycle threshold (C_q_) were inputted to all programs (Additional file 2: Table S2). For evaluation, the selected reference genes were listed based on their stability values (geNorm: M value; NormFinder: V_n_/V_n+1_; BestKeeper: Pearson’s r value; and comparative ΔC_q_: mean of SD value). Graphic were produced by GraphPad software (version 8.0). Descriptive statistics are shown as arithmetic mean values ± standard deviation (SD). The ranking sum for each gene is calculated by the summation of four respective rankings (Table 1).

### Results

**Fig. 1:**
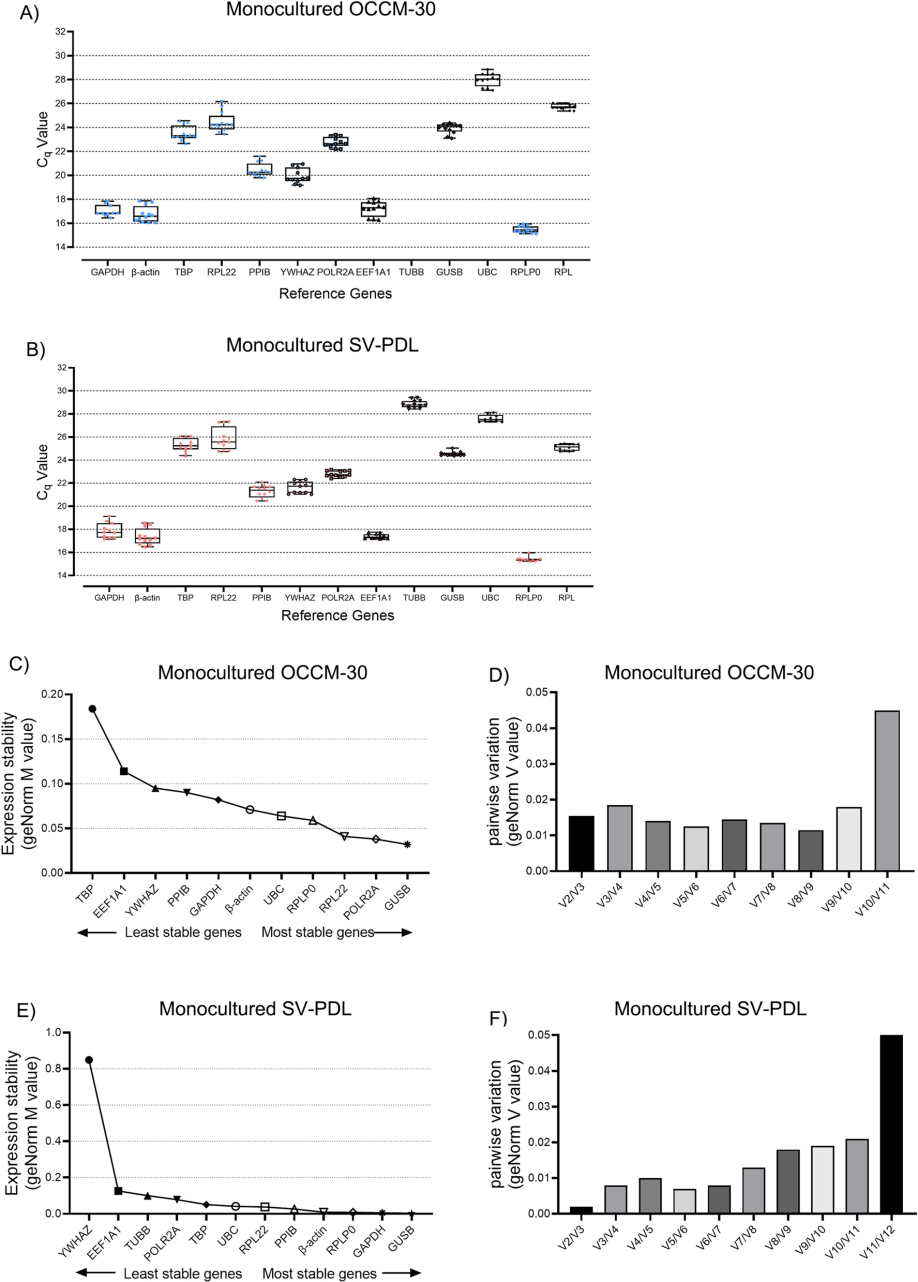
**A** C_q_ values are presented as quantification cycle (C_q_, n = 3) as second derivate maximum of the fluorescence curve and are inversely proportional to the amount of target mRNA within 1 μg of total RNA retrieved from the cementoblasts. C_q_ expression values of candidate reference genes, overall are for specimens without treatment (n = 13*3 duplication) in cementoblasts. **B** Expression levels of candidate reference genes in periodontal ligament cells (n = 13*3 duplication) without treatment. C_q_ values exported with identical threshold setting (mean of three technical replicates). Boxplots represent the median (central horizontal line), the interquartile range (IQR, 25/75 quartile, box) and the data range (whiskers) without outliners and extreme values. Outliers and extreme values are defined as C_q_ values more than 1.5 and 3 times the IQR apart from the upper/lower quartile and are denoted as circles and asterisms respectively. **C** On OCCM-30 cells, the geNorm analysis of the expression stability values (M value) of the 13 candidate reference genes, for which specific primers could be constructed. Average expression stability values of overall (pooled) specimens derived by stepwise exclusion of the least stable reference gene across all specimens and experiment conditions (n = 13*3 duplication). A smaller M value indicates a more stable expression. The most stable genes are on the right and the least stable genes are on the left. **D** Pairwise variation (V) of the 13 candidate reference genes calculated by geNorm to determine the suitable number of reference genes for OCCM-30 cells for RT-qPCR data normalization in overall studies (n = 13*3 duplication). The threshold used was 0.15. **E** On periodontal ligament cells, the geNorm analysis of the expression stability of the 13 candidate reference genes tested. Overall average expression stability values (M) derived by stepwise exclusion of the least stable reference gene across all specimens and experiment conditions. A higher M indicates a less gene expression. **F** Determination of the suitable number of reference genes for RT-qPCR data normalization on periodontal ligament cells. The geNorm calculation by the pairwise variation (V) indicates that V values lower than 0.15 indicated a sufficient normalization can be achieved

1. **Expression levels of candidate reference genes in mono cell culture**
  For OCCM-30 cells, as showed in Fig. 1A, the *β-actin*, *GAPDH*, *EEF1A1* and *RPLP0* are the candidate reference genes most abundantly expressed with C_q_ values below 2. The genes *TBP*, *RPL22*, *PPIB*, *YWHAZ*, *POLR2A*, *GUSB*, *UBC* and *RPL* are all moderately expressed with C_q_ values ranging from 20 to 30. Due to the cut-off applied, 10 out of 12 measurements for TUBB (C_q_ values 37.17) in the mono-cultured OCCM-30 dataset are removed from Fig. 1A.
  For SV-PDL cells, Fig. 1B shows that the *β-actin*, *GAPDH*, *EEF1A1* and *RPLP0* are the candidate reference genes most abundantly expressed with C_q_ values below 20. The genes *TBP*, *RPL22*, *PPIB*, *YWHAZ*, *POLR2A*, *TUBB*, *GUSB*, *UBC* and *RPL* are all moderately expressed with C_q_ values ranging from 20 to 30 in Fig. 1B.
2. **Stability analysis of candidate reference genes in mono cell culture system**
  For studies with monocultured cementoblasts, total ranking results in Table 1 show that *RPL22* is the least regulated reference gene in the preselected panel on OCCM-30 cells. Similarly, although *PPIB* is not as stable as *RPL22*, it ranks higher than *POLR2A* in all calculation in cementoblasts as showed in Fig. 1C.
  It is revealed that *GUSB* reached the best stability values on monocultured SV-PDL cells. The geNorm and the ΔC_q_ method in Fig. 1E show the same results. In this case, *GUSB* ranked highest in the comparison, but *GAPDH* was less stable compared to *RPLP0*.
  The geNorm analysis revealed that the use of two reference genes in this case *GUSB* and *GAPDH* for normalization in RT-qPCR is adequate for studies in monoculture of OCCM-30 cells (Fig. 1D) and SV-PDL cells (Fig. 1F). Notably, the output results of geNorm in the selection study showed that the M values of *TUBB* and *RPL* in OCCM-30 cells and *RPL* in SV-PDL cells were missing, indicating their exclusion for further analysis. The ranking order and the stability values calculated with the geNorm and NormFinder programs did not change when *TUBB* was excluded from the dataset.
  In concordance with the above given results, *PPIB*, *GUSB*, *RPLP0*, *POLR2A* and *RPL22* were selected as the most five stable reference genes based on ranking sum for further analysis in the direct cell–cell contact and co-culture system.
3. **Stability analysis of 5 chosen reference genes in direct cell–cell contact culture and co-culture system**

**Fig. 2:**
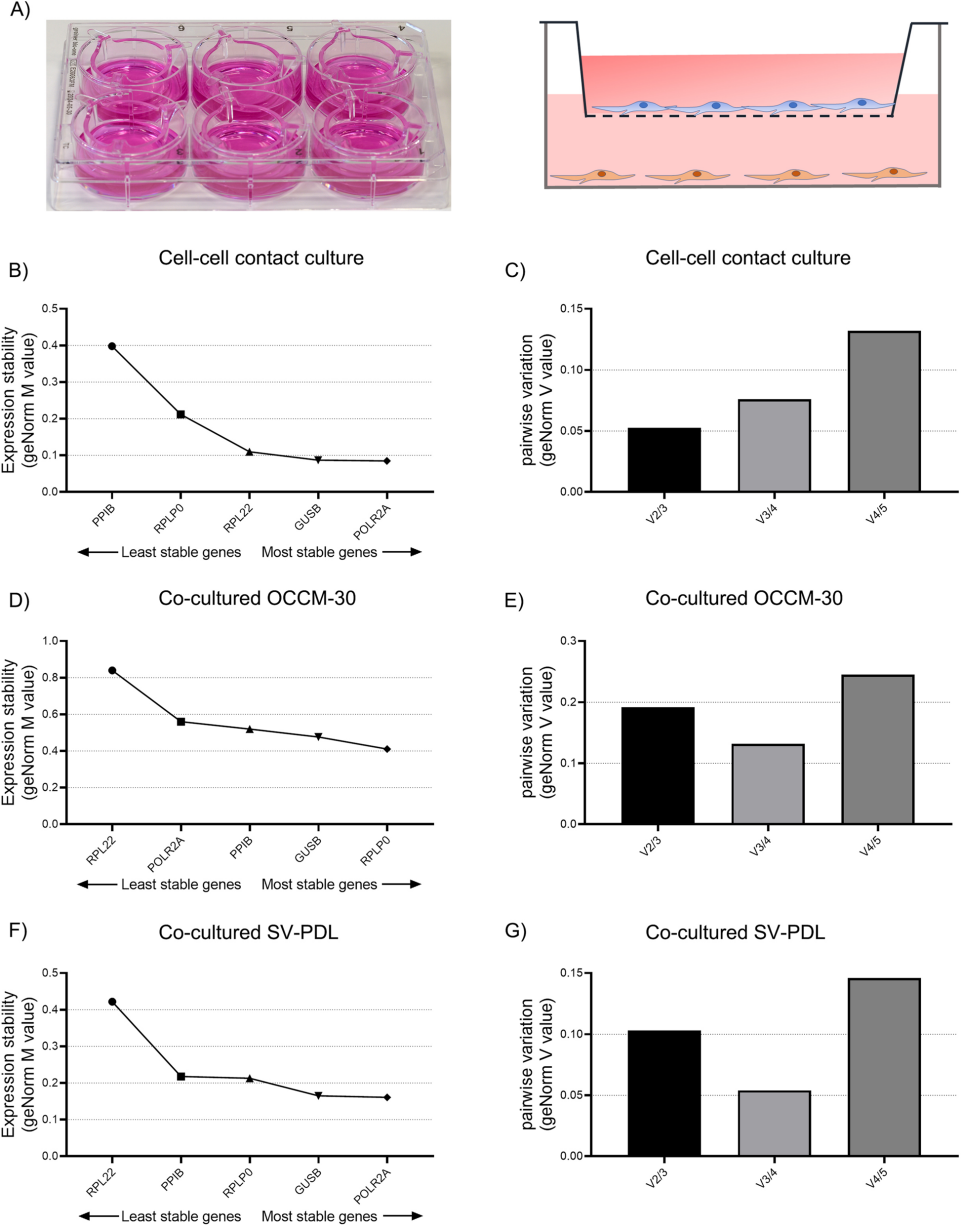
**A** Schematic representation of the co-culture system used in the experiment with SV-PDL cells plated on the upper insert and OCCM-30 cells in the lower well. **B**, **D**, **F** The stability of top four reference genes was assessed by geNorm (n = 15) for both the direct cell–cell contact culture system (**B**) and the co-culture system (**D**, **F**). Lower M value predicts higher stability. **C**, **E**, **G** The suitable number of reference genes was determined by geNorm for bothe direct cell–cell contact culture system (**C**) and the co-culture system (**E**, **G**). The value of V less than the recommended cut-off of 0.15 is attained with two reference genes

According to the single cell-culture results, the three highest ranking genes were selected for each cell line, respectively. These were *GUSB*, *POLR2A*, *RPLP0*, *RPL22* and *PPIB*. The entire ranking shows that *GUSB*, *POLR2A* and *RPLP0* were the least regulated reference genes when both cell lines were cultivated with direct contact. The suitable number of reference targets in the direct cell–cell contact experimental situation was 2. As such, the suitable normalization factor can be calculated as the geometric mean [21] of reference targets *GUSB* and *POLR2A* for the direct cell–cell contact culture system (Table 1, Fig. 2B, C).

As analyzed using different incubation times during the same day in the indirect co-culture systems, results showed that across three different time period, *PPIB*, *GUSB* and *RPLP0* have the best stability values for co-cultured OCCM-30 cells. The *PPIB*, *POLR2A* and *RPLP0* reached the best stability values for co-cultured SV-PDL cells. geNorm analysis shows that the suitable number of reference targets in this experimental situation was 2 and can be calculated as the geometric mean of reference targets *PPIB* and *POLR2A* (Table 1; Fig. 2D–G). Altogether, *PPIB* was the most stable reference gene for the co-culture system.

### Discussion

The in-vitro co-culture model is based on the location of OCCM-30 and SV-PDL cells which was subjected to mimicked specific conditions during OTM. The porosity membrane allows the cells to exchange soluble factors in the co-culture setup (Fig. 2A). Thus, the co-culture system seems reasonable to investigate the intercellular communication between these two cell lines of the periodontal compartment which become closer when orthodontic force is applied [10, 25]. However, reference gene selection is depending on the exact research questions and thus applies to both the experimental condition and the corresponding control, in this sense, the suitable reference genes in hypoxic- or orthodontic force-induced conditions need to be further investigated [25].

In the present work, from our analysis *PPIB* achieved the most stable results in all four algorithms methods for the use as a reference gene according to the comparison of all potential reference genes in co-culture system at different time point in the same day. This matches with previous research’s publication analyzing the combined dental, periodontal and alveolar bone tissue of rat which showed that *PPIB* and *YWHAZ* were the most stabile reference genes for RT-qPCR analysis in untreated rats with additional periodontitis [26]. *PPIB* is also reported to have the highest expression stability values and reliability on hPDLF subjected to static mechanical strain [27]. Besides *PPIB*, *GUSB* and *RPLP0* were ranked as the most stable reference genes for co-cultured OCCM-30 cells at different time periods. This is in concordance with the ranking in the control group, indicating that these three highest ranking genes are stably for the co-cultured OCCM-30 cells. Similarly, *PPIB*, *POLR2A* and *RPLP0* were recommended as the three most stable reference genes for co-cultured SV-PDL cells at different time points within the same day.

Direct cell–cell contact culture is more closely to the in-vivo condition that both cell types are cultivated together. However, it would be difficult to compare the gene expression in the direct cell–cell contact culture compared to the mono-cultured cells. Thus, for the purpose, discrimination of two types of cells using special surface markers assessed by flow cytometry would be more accurate to provide separate results of the different reference genes. Besides, for the indirect co-culture system, the C_q_ values expression of the reference genes may be changed dependent upon the specific placement of cells within the experimental setup. Although this study did not specifically refer to this issue, one might speculate that each cell type exerts different on the other when the position of two types of cells changed as shown in Fig. 2A. Furthermore, in the present proposed setup, the cells of two types in the insert and on the bottom of the wells are < 1 mm apart. Therefore, it might be necessary to investigate if the magnitude of gravity interferes with diffusion.

We concluded that *PPIB*, *GUSB* and *RPLP0* are the most stable reference genes for normalization in RT-qPCR studies using OCCM-30 cells in a co-culture system. *PPIB*, *POLR2A* and *RPLP0* were demonstrated to be the most reliable normalizers for SV-PDL cells used for RT-qPCR gene expression analysis in the co-culture system. The PPIB is an ideal reference and combination of *PPIB* and *POLR2A* for RT-qPCR experiments can improve the normalization in co-culture systems.

### Limitations

In direct cell–cell contact culture, the gene expression results should be considered as a mean of both cell types, thus it’s necessary to separate these two types of cells using FACS by their special membrane marker to provide separate results of reference genes. Different orthodontic induced conditions such as hypoxia and mechanical forces are not included in the stimulation, which needs to be further investigated.

## Supplementary Information

**Additional file 1**. MIQE checklist

**Additional file 2**. Input/output data for algorithms

## Data Availability

The datasets used and/or analysed during the current study are available from the corresponding author on reasonable request.

## Abbreviations

C_q_: Cycle threshold
FACS: Fluorescence-activated cell sorting
hPDLF: Human periodontal ligament fibroblasts
OCCM-30: Mouse cementoblast
OTM: Orthodontic tooth movement
PBS: Phosphate-buffer saline
RT-qPCR: Reverse transcriptase-quantitative polymerase chain reaction
SD: Standard deviation
SV-PDL: Periodontal ligament
