# Supplementary material for: Selection and validation of reference gene for RT-qPCR studies in co-culture system of mouse cementoblasts and periodontal ligament cells
Source: BMC Res Notes. 2022 Feb 15;15:57. doi: 10.1186/s13104-022-05948-x (PMC8845258; doi:10.1186/s13104-022-05948-x)
Supplement: Supplementary file 1 — Additional file 1. MIQE checklist [file 13104_2022_5948_MOESM1_ESM.docx]

Supplementary information to

**Selection and validation of reference gene for RT-PCR studies in co-culture system of mouse cementoblasts and periodontal ligament cells**

Supplementary Table 2. MIQE checklist

Reference: Bustin et al. (2010). BMC Mol Biol 11:74. http://dx.doi.org/10.1186/1471-2199-11-74

|  | **Details** | **Checklist** |
| --- | --- | --- |
| **Sample/Template** |  |  |
| Source | If cancer, was biopsy screened for adjacent normal tissue? | Immortalized murine mouse cementoblast (OCCM-30) cell line and immortalized murine periodontal ligament (SV-PDL) cells were provided by Prof. Martha J. Somerman (Laboratory of Oral Connective Tissue Biology, NIH, Bethesda, USA). |
| Method of preservation | Liquid N2/RNAlater/formalin | Liquid nitrogen (40% FBS, 15% DMSO, 45% cell culture medium (DMEM, 1% Penicillin/Streptomycin mixture)) freezing 1°C/min in cryo-box with isopropanol. |
| Storage time (if appropriate) | If using samples >6 months old | 6-12 months |
| Handling | Fresh/frozen/formalin | mRNA isolated directly from cultured cells; cell lysates from each sample were prepared using RNA lysis solution provided with the RNase Mini Kit (Qiagen, Germany) and cell lysates were stored at -80°C until further use. |
| Extraction method | TriZol/columns | Cell lysates were first passaged through on-column DNase columns (Qiagen) to shear genomic DNA. RNA extraction was done using RNase Mini Kit (Qiagen, Germany) as per instructions of the manufacturer including DNase digestion on the column. RNAse-Free DNase Set (Qiagen) was added and incubate at room temerature for 15 minutes. Purified total RNA was eluted with 30 µL using RNase/DNase-free water provided with the extraction kit. And total RNA preparations were stored at -80°C until further use. |
| RNA:DNA-free | Intron-spanning primers/no RT control | Most primers were exon-spanning including RPL22, PPIB, RPLP0, GUSB, β-actin, GAPDH, YWHAZ, EEF1A1, PRL. Intron-spanning includes POLR2A, TBP, UBC, TUBB. Additionally, DNase digestion (Qiagen) on the column was done according to the manufacturer's instructions. |
| Concentration | Nanodrop/ribogreen/microfluidics | Purity and concentration was determined photometrically (Nanodrop 2000, Thermo Fisher Scientific, USA). A ratio of A_260/280_>1.8 indicated protein-free RNA preparations. |
| RNA: integrity | Microfluidics/3':5' assay | No. |
| Inhibition-free | Method of testing | Serial dilution of cDNA; see below "PCR efficiency". |
| **Assay optimisation/validation** | |  |
| Accession number | RefSeq XX_1234567 | RPL22 (NC_000070.6), PPIB (NC_000075.6), POLR2A (NC_000077.6), RPLP0 (NT_078458.7), GUSB (NC_000071.6), β-actin (NC_000071.6), TBP (NC_000083.6), UBC (NC_000071.6), GAPDH (NC_000072.6), YWHAZ (NC_000081.6), EEF1A1 (NC_000075.6), RPL (NM_012535) and TUBB (NC_000083.6) |
| Amplicon details | Exon location, amplicon size | RPL22 (4:152332921-152333013, 63 bp), PPIB (9:66060262-66060399, 108 bp), POLR2A (11:69737466-69739478, 109 bp), RPLP0 (5:115563376-115563470, 65 bp), GUSB (5:130000361-130000469, 79 bp), β-actin (5:142904472-142904610, 109 bp), TBP (17:15503049-15504325, 102 bp), UBC (5:125388154-125389944, 116 bp), GAPDH (6:125162278-125162382, 75 bp), YWHAZ (15:36790882-36790971, 60 bp), EEF1A1 (9:78480599-78481641, 62 bp), RPL (17:44640025-44640423, 95 bp) and TUBB (17:57081655-57086020, 92 bp) |
| Primer sequence | Even if previously published | Sequence of primers purchased from Bio-Rad was disclosed upon purchase. Unique Assay ID (Bio-Rad) are listed:  RPL22 (qMmuCED0040736), PPIB (qMmuCED0047854), POLR2A (qMmuCID0005230), RPLP0 (qMmuCED0040751), GUSB (qMmuCED0004608), β-actin (qMmuCED0027505), TBP (qMmuCID0040542), UBC (qMmuCID0021036), GAPDH (qMmuCED0027497), YWHAZ (qMmuCED0027504), EEF1A1 (qMmuCED0025136), RPL (qRnoCED0016171^[[1]](#footnote-1)^) and TUBB (qMmuCID0026910). |
| *Probe sequence** | Identify LNA or other substitutions | No probes were used. |
| *In silico* | BLAST/Primer-BLAST/m-fold | Not applicable for commercial primer (Bio-Rad) |
| empirical | Primer concentration/annealing temperature | Not applicable for commercial primer (Bio-Rad) |
| Priming conditions | Oligo-dT/random/combination/target-specific | cDNA synthesis was done using the iScrip^TM^ cDNA Synthesis Kit (Bio-Rad) following precisely the manufacturers instructions using 1000 ng total RNA for each reaction. For qPCR target-specific primers were used as indicated in Table 1. |
| PCR efficiency | Dilution curve | qPCR assays output the construction of a standard curve, enabling the determination of the efficiency. For each gene qPCR was performed as given in the manuscript. Bio-Rad CFX Manager Software 3.1 (Bio-Rad) was used to analyze qPCR reactions including standard curves: RPL22 (efficiency: 1.907; R2: 0.00931; slope: -3.450; Y intercept: 36.89); PPIB (efficiency: 1.905; R2: 0.00485; slope: -3.672; Y intercept: 41.06); POLR2A (efficiency: 1.922; R2: 0.00773; slope: -3.471; Y intercept: 31.90); RPLP0 (efficiency: 1.905; R2: 0.0130; slope: -3.671; Y intercept: 38.71); GUSB (efficiency: 1.905; R2: 0.00876; slope: -3.565; Y intercept: 33.28); β-actin (efficiency: 1.856; R2: 0.0169; slope: -3.612; Y intercept: 37.04); TBP (efficiency: 1.908; R2: 0.0303; slope: -3.662; Y intercept: 37.98); UBC (efficiency: 1.795; R2: 0.00725; slope: -3.961; Y intercept: 29.12); GAPDH (efficiency: 1.909; R2: 0.00843; slope: -3.524; Y intercept: 36.63); YWHAZ (efficiency: 1.916; R2: 0.00923; slope: -3.321; Y intercept: 37.93); EEF1A1 (efficiency: 1.879; R2: 0.00718; slope: -3.944; Y intercept: 36.91); RPL (efficiency: 1.919; R2: 0.00741; slope: -3.934; Y intercept: 36.43); and TUBB (efficiency: 1.713; R2: 0.00963; slope: -3.684; Y intercept: 37.53).  Primer specificity was controlled by melting curve analysis after qRT-PCR cycles analysis that were carried out between 50°C and 95°C with a plate read every 0.5°C increment after holding the temperature for 5 seconds with continuous fluorescence acquisition. Detection was performed using the CFX96TM Real-Time System (C1000TM Thermal Cycler, Bio-Rad). The melting curves were created by plotting the differential fluorescence intensity vs. the temperature by Bio-Rad CFX Manager Software 3.1 (Bio-Rad). All of the reactions were performed in at least triplicate. |
| Linear dynamic range | Spanning unknown targets | Linear dynamic range was automatically appointed by qPCR analysing software. |
| Limits of detection | LOD detection/accurate quantification | LOD was automatically appointed by qPCR analysing software. |
| Intra-assay variation | Copy numbers not Cq | Each gene was assayed on individual plates. |
| **RT/PCR** |  |  |
| Protocols | Detailed description, concentrations, volumes | qPCR was carried out in a CFX96^TM^ Real-Time System Cycler (Bio-Rad) and analyzed using Bio-Rad CFX Manager version 3.1 software (Bio-Rad) using the SsoAdvanced^TM^ Universal SYBR^@^ Green Supermix (1723271, Bio-Rad) according to the manufacturer's protocol using 1 µL of the cDNA and 1 µL of target Bio-Rad primer. The supermix contains dUTP and Uracil-DNA glycosylase (UDG) to prevent carryover contamination between reactions. The PCR cycling protocol was as follows: reaction conditions comprised 40 cycles repeat and were denatured initially for 15 seconds at 95°C, subsequently, the cycling stage was performed at 95°C for 15 seconds and 1 minute of amplification at 60°C. Primer specificity was controlled by melting curve analysis after qRT-PCR cycles analysis that were carried out between 50°C and 95°C with a plate read every 0.5°C increment after holding the temperature for 5 seconds with continuous fluorescence acquisition. Detection was performed using the CFX96^TM^ Real-Time System (C1000TM Thermal Cycler, Bio-Rad). The melting curves were created by plotting the differential fluorescence intensity vs. the temperature by Bio-Rad CFX Manager Software 3.1 (Bio-Rad). All of the reactions were performed in at least triplicate. |
| Reagents | Supplier, Lot number | Primers (purchased from Bio-Rad, Germany); Universal SYBR^@^ Green Supermix (purchased from Bio-Rad, Germany) |
| Duplicate RT | ΔCq | No, but each biological sample was repeated minimum two independent times. |
| NTC | Cq & melt curves | Yes |
| NAC | ΔCq beginning:end of qPCR | No, since no probes were used. |
| Positive control | Inter-run calibrators | No, for each gene all samples were analyzed together on one plate. |
| **Data analysis** |  |  |
| Specialist software | e.g., QBAsePlus | GraphPad software (version 8.0, USA) |
| Statistical justification | e.g., biological replicates | For each day-culture-combination three biological replicas were used (see experimtal setup outlined in Material and Methods). All cDNA samples were measured twice, giving a total of six PCR amplifications for each gene/culture/time combination. |
| Transparent, validated normalisation | e.g., GeNorm summary | From the calculation and ranking of four methods (geNorm (qBase+, Biogazelle), NormFinder (version 0.953), BestKeeper (version 1) and Comparative ΔC_q_ method), the detals of results are shown in the results section from a panel of thirteen different reference genes. |

1. The sequence from the rat RPL primer (qRnoCED0016171) were blaseted and found out that the sequence between rat and mouse is partially same [Gene: Prl3b1 (Prolactin family 3, subfamily b, member 1) Rattus norvegicus]. Moreover, in the experiment the rat RPL primer produced stable melting curves in the present two cell lines. Thus, the rat RPL primer was used in the present experimental setup. [↑](#footnote-ref-1)
