# Supplementary material for: Selection and validation of reference gene for RT-qPCR studies in co-culture system of mouse cementoblasts and periodontal ligament cells
Source: BMC Res Notes. 2022 Feb 15;15:57. doi: 10.1186/s13104-022-05948-x (PMC8845258; doi:10.1186/s13104-022-05948-x)
Supplement: Supplementary file 2 — Additional file 2. Input/output data for algorithms [file 13104_2022_5948_MOESM2_ESM.docx]

Supplementary information to

**Selection and validation of reference gene for RT-PCR studies in co-culture system of mouse cementoblasts and periodontal ligament cells**

Supplementary Table 3. Input/output data for algorithms

Part 1. Stability assessment by four algorithms

Part 2. Input data for four algorithms

Part 3. Output data of four algorithms

Part 4. Original geNorm output data

**Part 1. Stability assessment of four algorithms**

geNorm analysis: The geNorm calculates the average pairwise C_q_ variation which is given as expression stability M. The lowest M values of genes indicate a smaller average pairwise variation in gene expression and should thus be included for normalization. In our validation study, geNorm was used to calculate the optimal combination of reference genes by the average pairwise variation (V_n_/V_n+1_) between n and n+1 candidate genes, achieving to identify the minimal number of reference genes for reliable normalization with a cut-off value of 0.15 [21].

NormFinder analysis: The NormFinder algorithm can differentiate intragroup variation from intergroup variation for normalization factor calculation by creating a combined stability value for each candidate reference gene using a model-based approach with lower stability values associated with higher expression stability [22].

BestKeeper analysis: BestKeeper were able to determine stability depending on the standard deviation (SD) of C_q_ means of every candidate reference gene as well as Pearson’s correlation coefficient (r value) by pairwise bivariate correlations of C_q_ values of every gene. Higher r values are interpreted as more stably expressed genes [23].

Comparative ΔC_q_ analysis: The comparative ΔC_q_ method compares the relative expression of pair of genes within each sample and executed by manual calculations [24]. This algorithm compares the relative expression of reference gens within groups of biological replicates based on the mean SD [25]. Thus, a lower SD indicates a more stable gene expression.

**Part 2. Input data for four algorithms**

| ***monocultured cementoblasts (OCCM-30)*** | | | | | | | | | | | | |
| --- | --- | --- | --- | --- | --- | --- | --- | --- | --- | --- | --- | --- |
| Reference genes | C_q_ values | | | | | | | | | | | |
| GAPDH | 20.40 | 24.20 | 16.45 | 16.77 | 16.64 | 16.85 | 16.82 | 16.83 | 16.85 | 17.84 | 17.47 | 17.70 |
| β-actin | 16.24 | 16.05 | 16.07 | 16.81 | 16.73 | 16.65 | 16.40 | 16.52 | 16.10 | 17.65 | 17.76 | 17.87 |
| TBP | 23.06 | 22.90 | 22.66 | 23.37 | 23.27 | 23.29 | 23.33 | 23.48 | 23.23 | 24.44 | 24.39 | 24.55 |
| RPL | 23.77 | 23.56 | 23.43 | 24.21 | 24.31 | 24.22 | 24.25 | 24.20 | 24.05 | 25.21 | 25.72 | 26.16 |
| PPIB | 19.99 | 19.91 | 19.81 | 20.25 | 20.22 | 20.24 | 20.19 | 20.33 | 20.41 | 21.18 | 21.22 | 21.59 |
| YWHAZ | 19.53 | 19.26 | 19.17 | 19.65 | 19.74 | 19.78 | 19.66 | 20.21 | 27.99 | 20.94 | 20.66 | 20.85 |
| POLR2A | 23.39 | 23.27 | 23.30 | 22.79 | 22.55 | 22.63 | 22.62 | 22.52 | 23.05 | 22.20 | 22.19 | 22.42 |
| EEF1A1 | 18.06 | 17.77 | 17.71 | 17.21 | 17.82 | 17.32 | 17.42 | 17.17 | 17.22 | 16.32 | 16.26 | 16.33 |
| TUBB^[[1]](#footnote-1)^ |  |  |  |  |  |  |  | 36.87 |  |  | 37.46 |  |
| GUSB | 24.21 | 24.38 | 24.25 | 24.26 | 24.15 | 24.21 | 23.90 | 23.79 | 23.99 | 23.21 | 23.10 | 23.60 |
| UBC | 28.47 | 28.15 | 28.01 | 28.45 | 28.42 | 28.85 | 27.94 | 28.03 | 27.93 | 27.10 | 27.26 | 27.22 |
| RPLP0 | 15.94 | 15.84 | 15.76 | 15.41 | 15.50 | 15.46 | 15.34 | 15.22 | 15.33 | 15.12 | 15.15 | 15.71 |
| RPL22 | 25.75 | 25.38 | 25.65 | 25.68 | 25.39 | 25.59 | 25.67 | 25.87 | 26.02 | 26.00 | 25.99 | 26.04 |
| ***monocultured periodontal ligament cells (SV-PDL)*** | | | | | | | | | | | | |
| Reference genes | C_q_ values | | | | | | | | | | | |
| GAPDH^[[2]](#footnote-2)^ |  | 17.73 | 17.28 | 19.13 | 18.52 | 18.70 | 17.73 | 18.07 | 17.92 | 17.39 | 17.15 | 17.26 |
| β-actin | 16.71 | 16.61 | 16.49 | 18.28 | 18.42 | 18.54 | 17.24 | 17.43 | 17.07 | 17.02 | 17.20 | 17.35 |
| TBP | 24.88 | 24.61 | 24.38 | 26.08 | 26.02 | 26.08 | 25.10 | 25.34 | 25.47 | 25.10 | 25.14 | 25.58 |
| RPL | 24.84 | 24.77 | 24.73 | 27.29 | 27.31 | 27.24 | 25.52 | 25.86 | 26.01 | 25.27 | 25.44 | 25.64 |
| PPIB | 20.67 | 20.49 | 20.46 | 21.69 | 21.54 | 21.55 | 21.68 | 21.79 | 22.09 | 21.26 | 21.06 | 21.07 |
| YWHAZ^[[3]](#footnote-3)^ | 21.18 | 21.04 | 21.05 | 22.31 | 22.13 | 22.33 | 21.99 | 21.75 | 21.81 | 21.22 |  | 21.18 |
| POLR2A | 22.40 | 22.53 | 22.58 | 22.66 | 22.74 | 22.73 | 23.16 | 23.09 | 23.06 | 22.99 | 22.69 | 23.09 |
| EEF1A1 | 17.40 | 17.22 | 17.19 | 17.23 | 17.15 | 17.21 | 17.46 | 17.38 | 17.25 | 17.69 | 17.61 | 17.75 |
| TUBB | 28.41 | 29.37 | 29.41 | 28.82 | 28.81 | 29.08 | 28.54 | 28.72 | 28.74 | 28.47 | 28.67 | 29.14 |
| GUSB | 24.51 | 24.72 | 24.63 | 24.43 | 24.40 | 24.52 | 24.40 | 24.36 | 24.50 | 25.03 | 24.42 | 24.63 |
| UBC^[[4]](#footnote-4)^ | 27.52 | 31.03 |  |  | 28.04 | 27.80 | 27.36 | 27.31 | 27.30 | 27.51 | 27.66 | 28.12 |
| RPLP0 | 15.37 | 15.33 | 15.21 | 15.22 | 15.32 | 15.96 | 15.39 | 15.29 | 15.44 | 15.48 | 15.25 | 15.44 |
| RPL22 | 25.43 | 25.39 | 25.37 | 24.85 | 24.97 | 25.07 | 24.74 | 24.74 | 24.80 | 25.23 | 25.38 | 25.38 |

| ***Direct cell-cell contact cultured of OCCM-30 and SV-PDL*** | | | | | | | | | |
| --- | --- | --- | --- | --- | --- | --- | --- | --- | --- |
| 5 chosen reference genes | C_q_ values | | | | | | | | |
|  | 0 h | | | 12 h | | | 24 h | | |
| RPL22 | 30.05 | 27.70 | 27.49 | 27.45 | 28.86 | 28.02 | 27.92 | 27.77 | 27.26 |
|  | 29.75 | 27.75 | 27.45 | 27.57 | 28.69 | 27.75 | 27.72 | 27.25 | 26.88 |
| PPIB | 22.55 | 21.66 | 21.43 | 21.09 | 26.37 | 21.52 | 22.15 | 21.44 | 20.61 |
|  | 22.77 | 21.93 | 21.84 | 21.21 | 24.76 | 21.73 | 22.48 | 21.14 | 20.51 |
| POLR2A | 26.57 | 25.14 | 25.38 | 24.51 | 25.98 | 24.70 | 25.04 | 24.59 | 24.23 |
|  | 26.44 | 25.16 | 25.51 | 24.39 | 26.04 | 24.88 | 25.10 | 24.67 | 24.04 |
| GUSB | 27.28 | 26.27 | 26.82 | 25.75 | 26.98 | 26.19 | 26.98 | 25.66 | 24.91 |
|  | 27.70 | 26.43 | 26.93 | 25.67 | 26.89 | 26.20 | 26.54 | 25.93 | 25.00 |
| RPLP0 | 15.65 | 15.73 | 16.51 | 15.39 | 15.96 | 15.58 | 16.13 | 15.47 | 15.13 |
|  | 15.94 | 16.21 | 16.04 | 15.07 | 15.93 | 15.50 | 16.41 | 15.28 | 14.92 |

| ***Co-cultured OCCM-30 control and with 3 indicated time*** | | | | | | | | | |
| --- | --- | --- | --- | --- | --- | --- | --- | --- | --- |
| 5 chosen reference genes | C_q_ values | | | | | | | | |
|  | 0 h | | | 12 h | | | 24 h | | |
| RPL22 | 27.16 | 27.01 | 30.52 | 28.06 | 28.07 | 28.07 | 25.94 | 26.23 | 27.19 |
|  | 27.20 | 26.93 | 30.35 | 28.09 | 27.69 | 27.99 | 25.74 | 26.04 | 26.81 |
| PPIB | 20.97 | 21.26 | 23.18 | 22.63 | 22.77 | 23.07 | 21.51 | 21.96 | 22.81 |
|  | 21.12 | 21.45 | 23.50 | 23.07 | 22.92 | 23.21 | 21.49 | 22.06 | 22.89 |
| POLR2A | 27.11 | 25.00 | 26.65 | 25.40 | 26.05 | 25.89 | 25.24 | 25.42 | 26.96 |
|  | 24.53 | 24.82 | 26.65 | 25.45 | 25.23 | 25.69 | 25.31 | 25.34 | 26.07 |
| GUSB | 26.21 | 26.21 | 28.44 | 27.50 | 27.42 | 27.58 | 27.67 | 28.10 | 28.46 |
|  | 26.43 | 26.51 | 28.45 | 27.38 | 27.40 | 27.48 | 27.86 | 28.00 | 28.51 |
| RPLP0 | 15.59 | 16.49 | 16.19 | 17.57 | 17.10 | 16.70 | 16.79 | 16.65 | 17.78 |
|  | 15.73 | 16.24 | 16.09 | 17.39 | 17.03 | 16.52 | 16.37 | 17.16 | 17.28 |
|  | | | | | | | | | |
| ***Co-cultured SV-PDL control and with 3 indicated time*** | | | | | | | | | |
| 5 chosen reference genes | C_q_ values | | | | | | | | |
|  | 0 h | | | 12 h | | | 24 h | | |
| RPL22 | 28.08 | 28.78 | 29.61 | 27.61 | 27.16 | 27.60 | 27.89 | 27.15 | 28.03 |
|  | 28.04 | 28.60 | 29.53 | 27.48 | 26.86 | 27.54 | 27.77 | 27.22 | 27.69 |
| PPIB | 21.12 | 21.61 | 22.34 | 21.42 | 20.76 | 21.52 | 21.60 | 21.38 | 21.91 |
|  | 21.16 | 21.95 | 22.42 | 21.61 | 20.96 | 21.87 | 21.83 | 21.34 | 21.62 |
| POLR2A | 24.46 | 25.09 | 26.21 | 25.37 | 24.71 | 26.18 | 26.08 | 25.33 | 25.59 |
|  | 24.45 | 25.08 | 26.27 | 25.59 | 24.72 | 25.73 | 25.67 | 25.55 | 25.62 |
| GUSB | 25.39 | 25.87 | 27.16 | 26.71 | 25.98 | 26.88 | 27.13 | 26.28 | 27.00 |
|  | 25.73 | 26.37 | 27.56 | 26.77 | 25.94 | 26.78 | 26.76 | 26.34 | 26.91 |
| RPLP0 | 15.62 | 15.85 | 15.91 | 15.42 | 15.39 | 15.53 | 15.45 | 15.58 | 16.01 |
|  | 15.28 | 16.06 | 16.00 | 15.35 | 15.25 | 15.59 | 15.33 | 15.54 | 15.77 |

**Part 3. Output data of four algorithms**

| ***monocultured cementoblasts (OCCM-30)*** | | | |
| --- | --- | --- | --- |
| geNorm | NormFinder | Comparative ΔCq | BestKeeper |
| 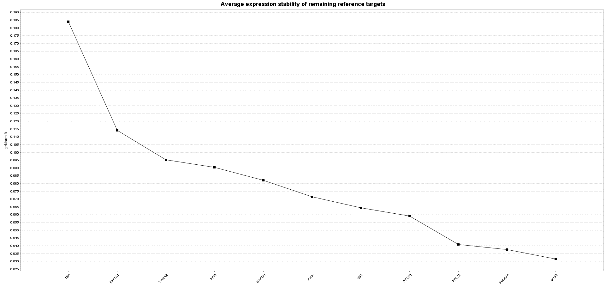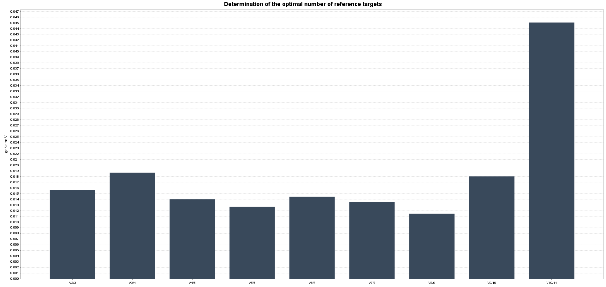 | 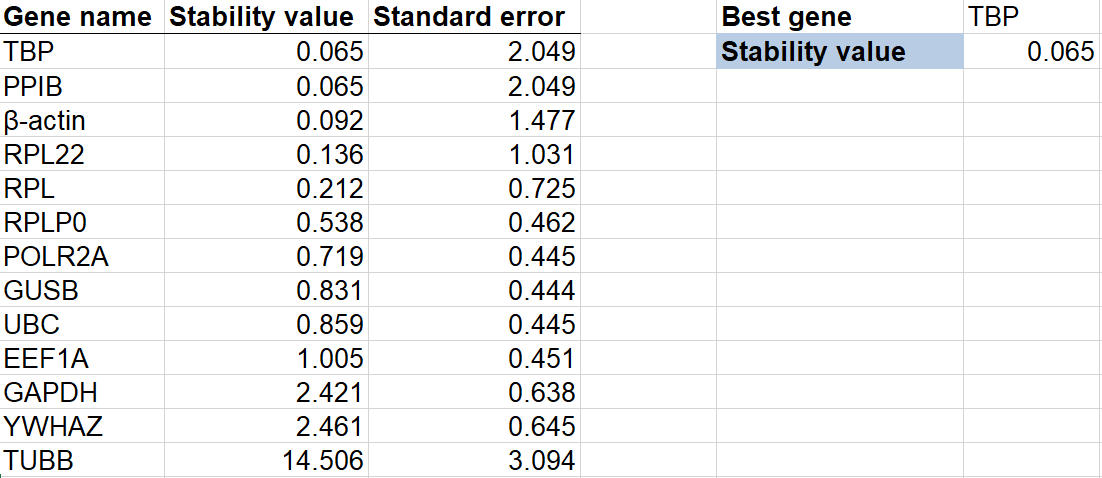 | 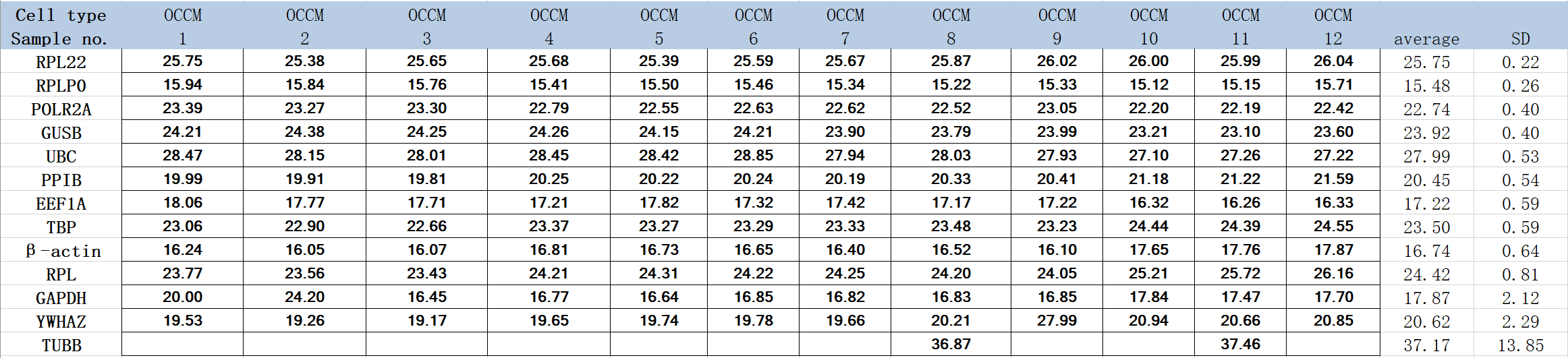 | 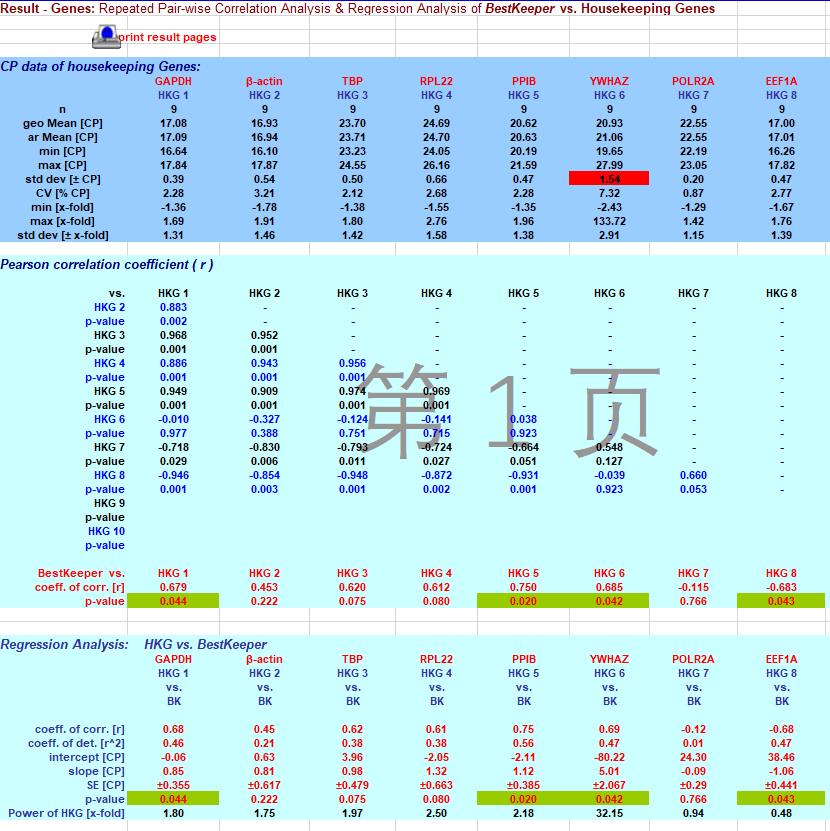 |
| 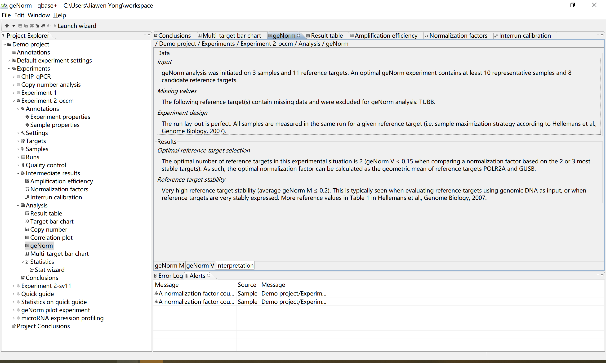 |  |  | 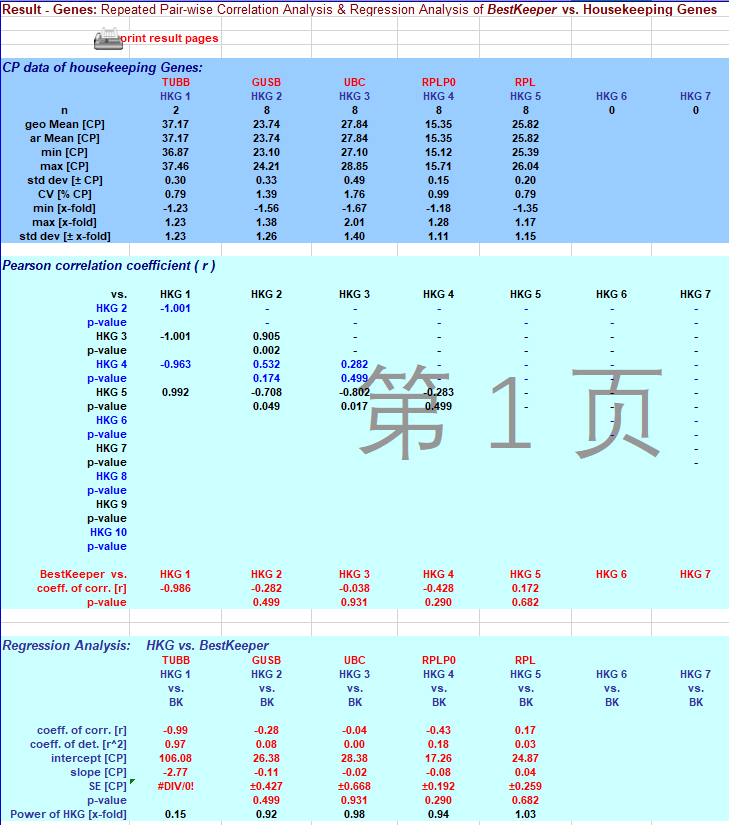 |

| ***monocultured periodontal ligament cells (SV-PDL)*** | | | |
| --- | --- | --- | --- |
| geNorm | NormFinder | Comparative ΔCq | BestKeeper |
| 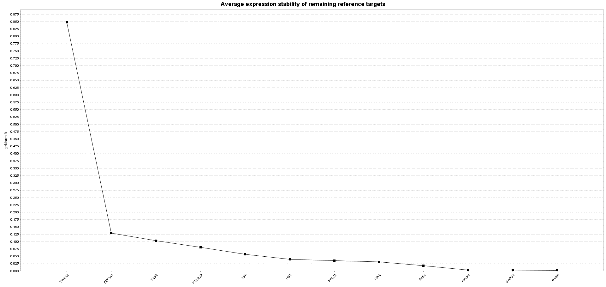  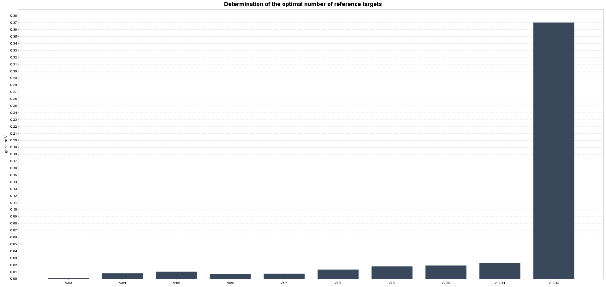 | 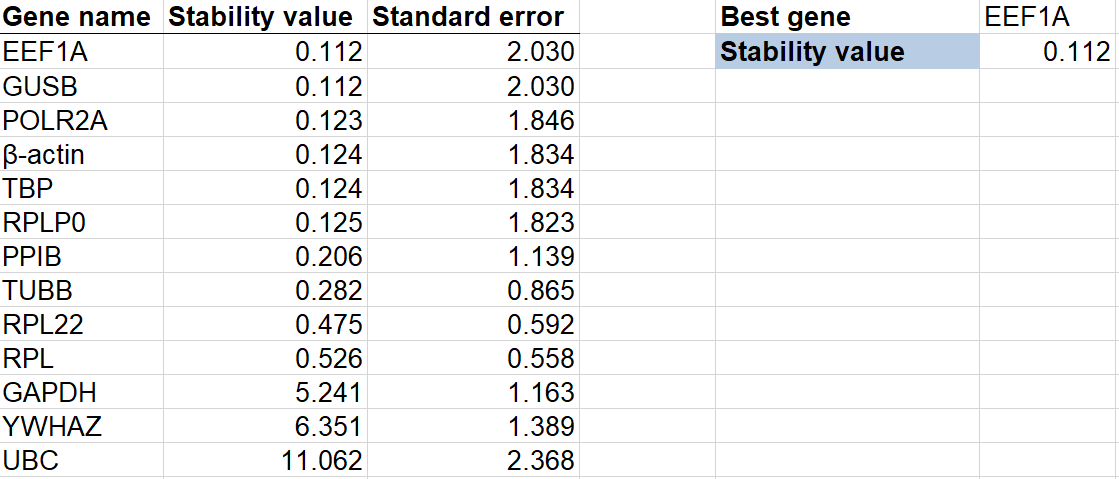 | 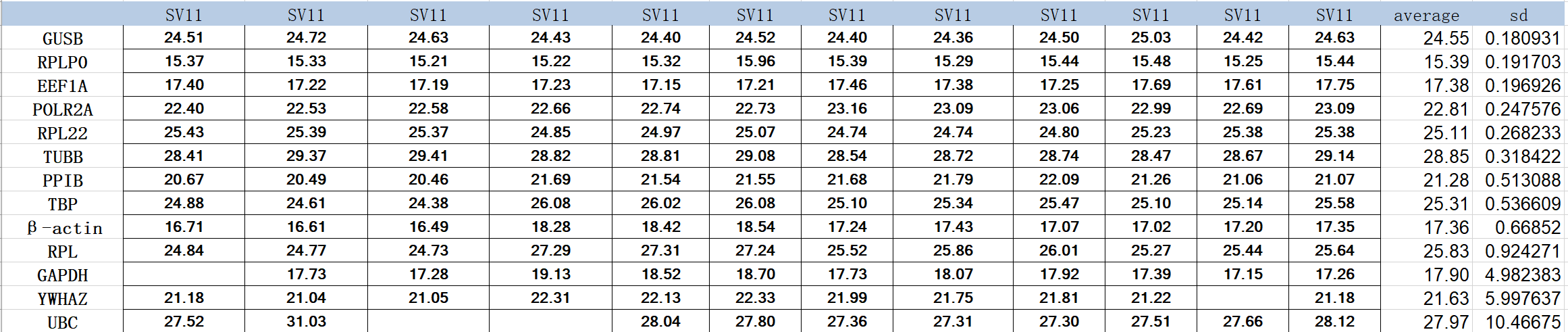 | 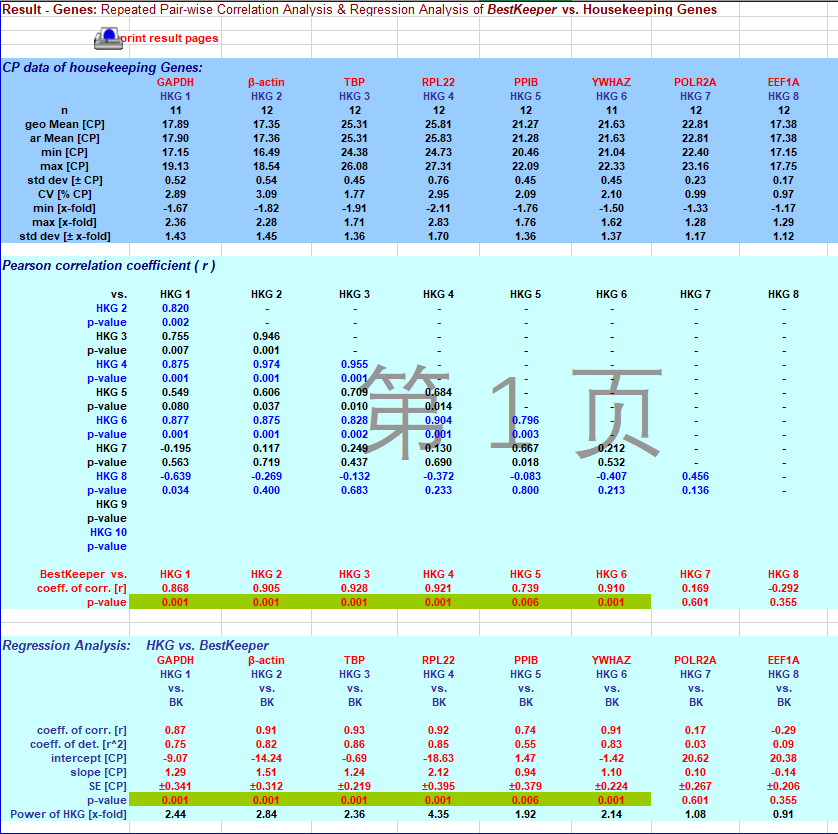 |
| 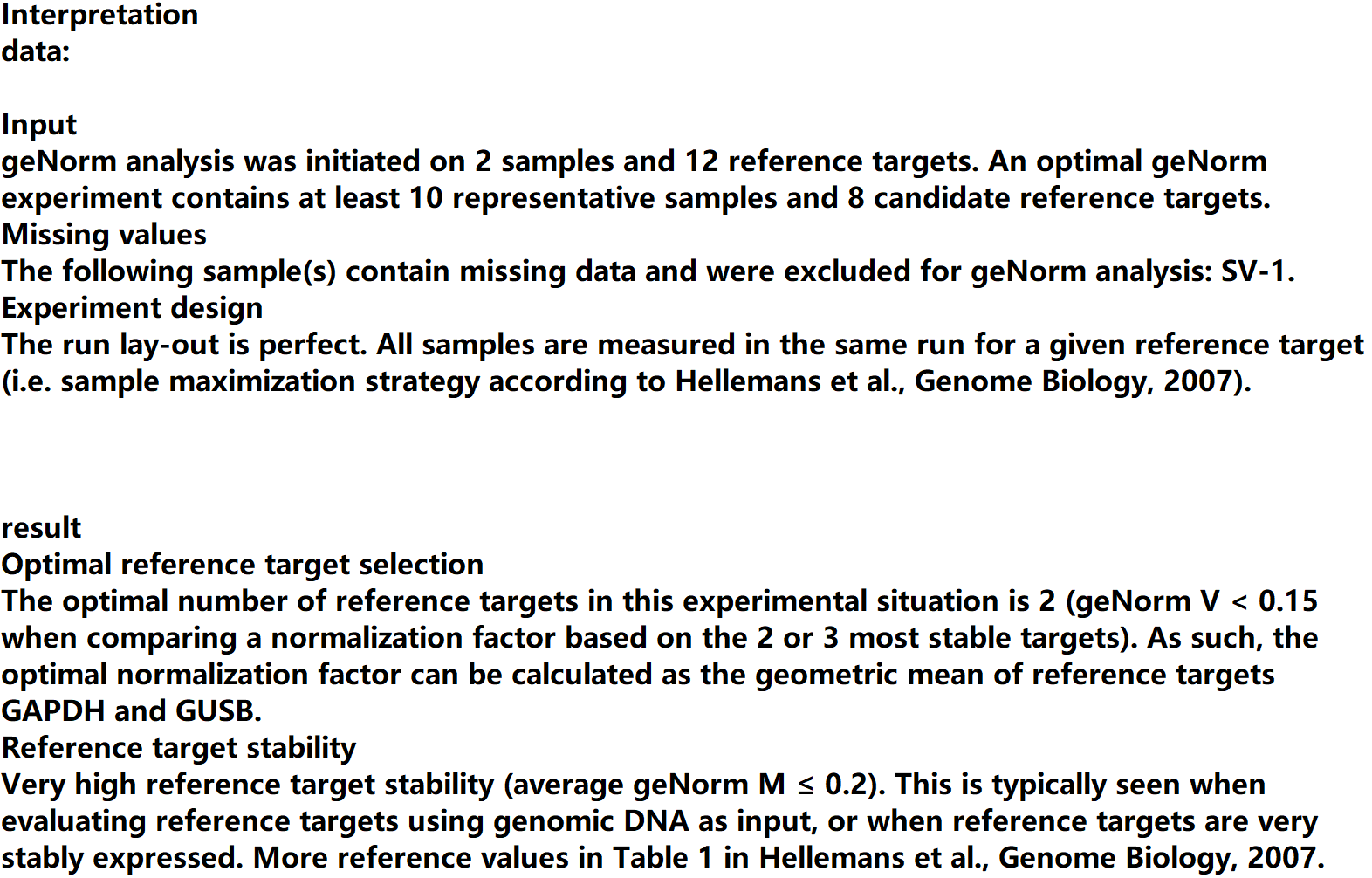 |  |  | 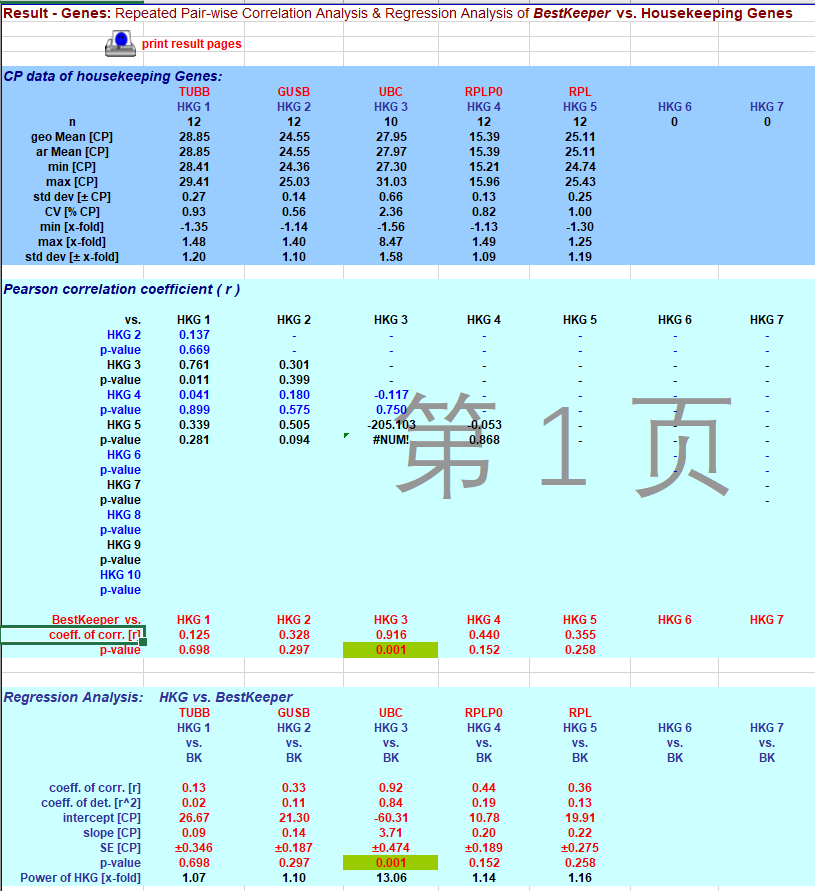 |

| ***Direct cell-cell contact cultured of OCCM-30 and SV-PDL*** | | | |
| --- | --- | --- | --- |
| geNorm | NormFinder | Comparative ΔCq | BestKeeper |
| 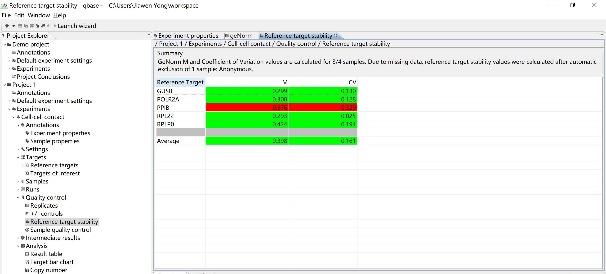  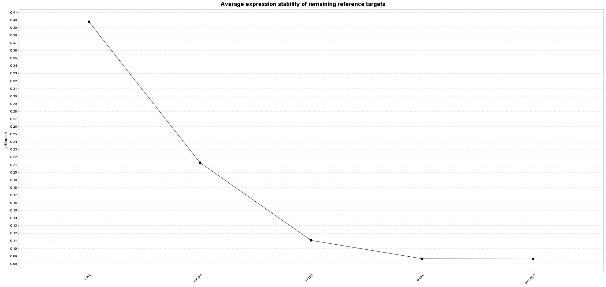 | 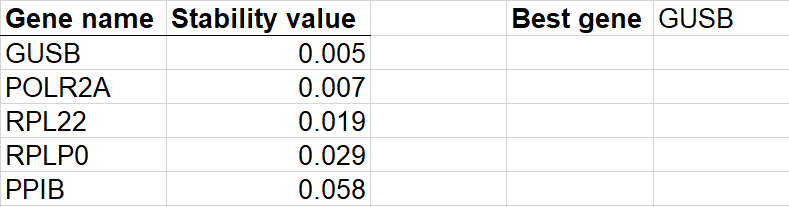 | 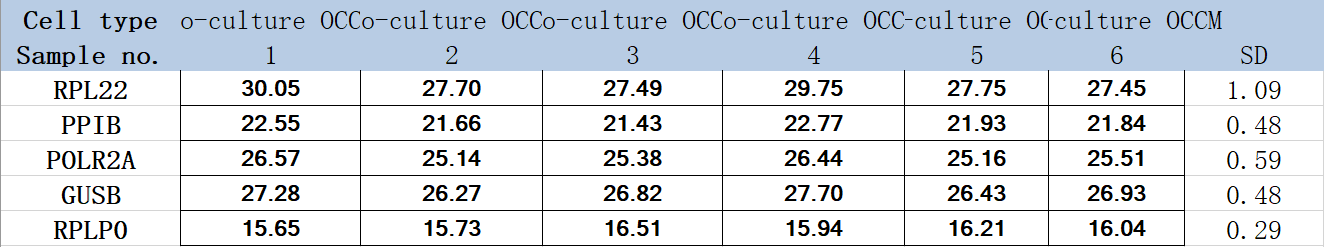 | 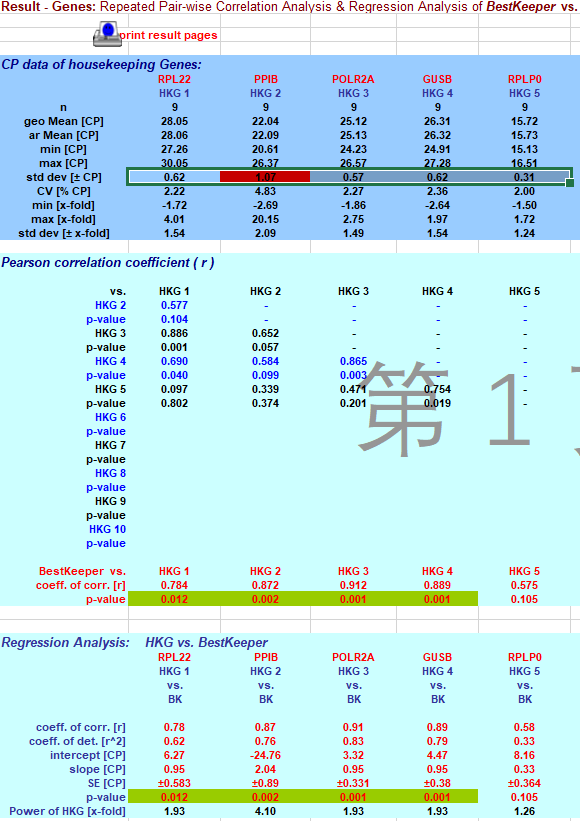 |
| 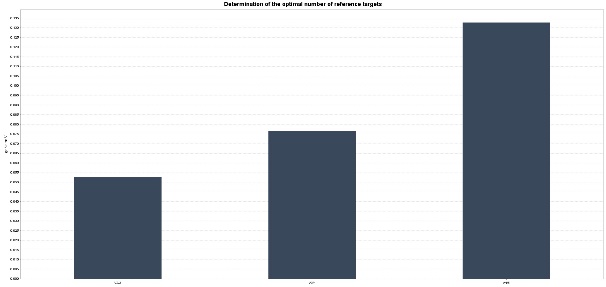 |  |  |  |
| 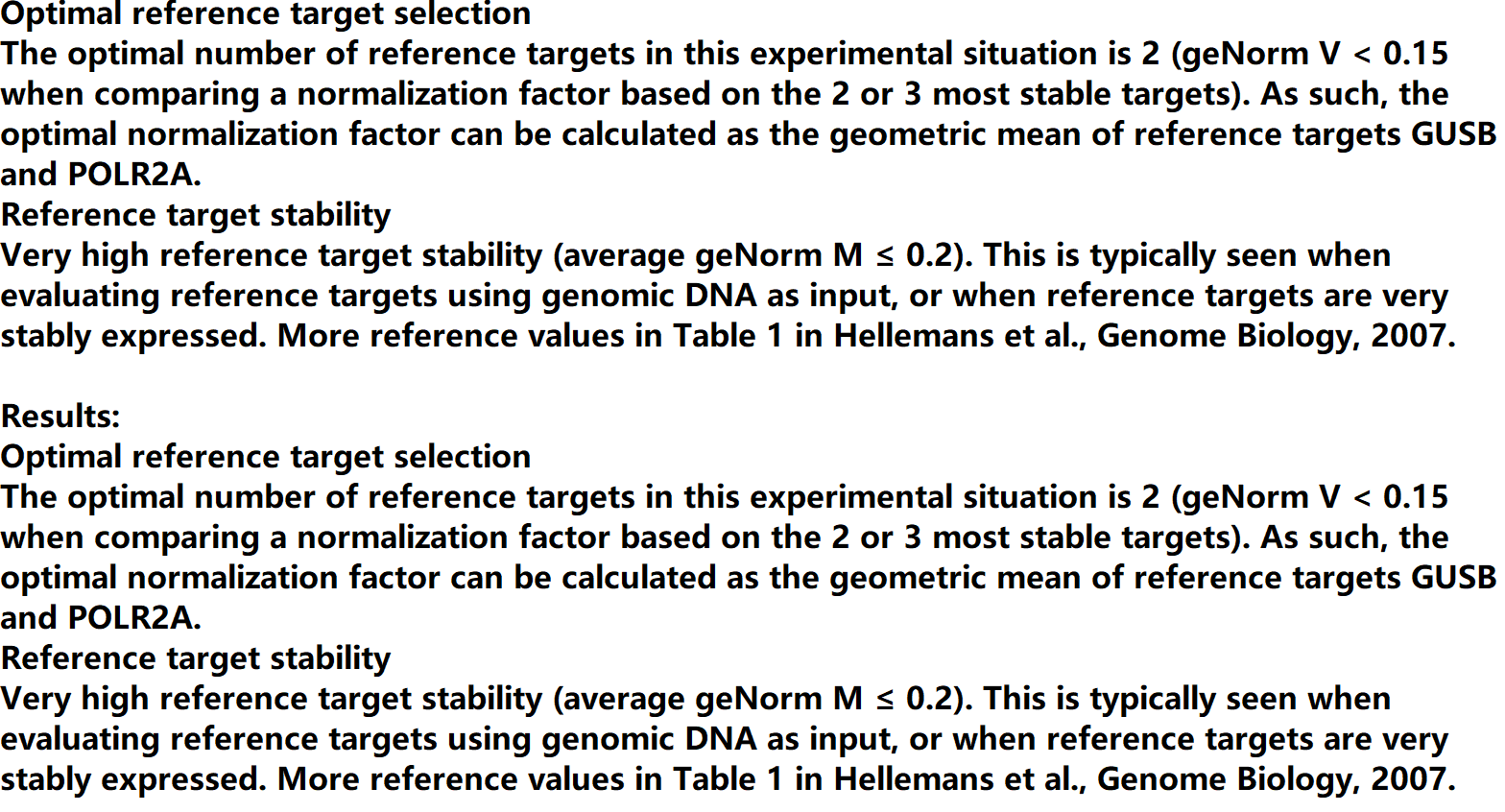 |  |  |  |

| ***Co-cultured OCCM-30 control and with 3 indicated time*** | | | |
| --- | --- | --- | --- |
| geNorm | NormFinder | Comparative ΔCq | BestKeeper |
| ***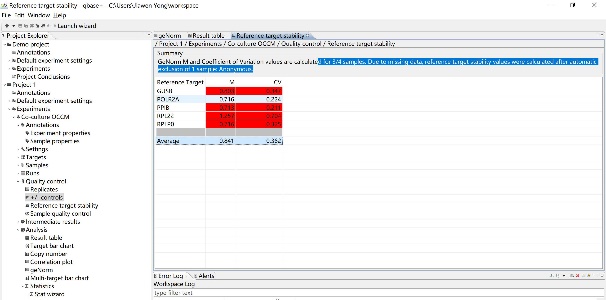***  ***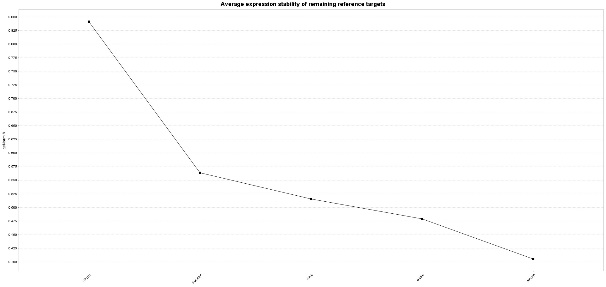*** | Co-cultured OCCM-30 control  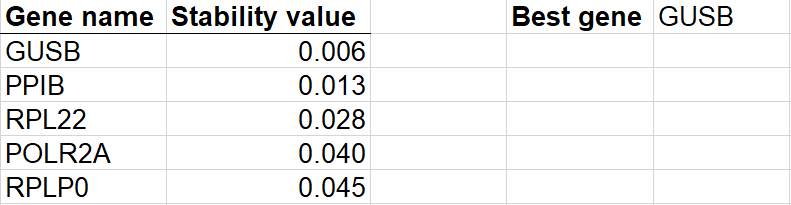 | Co-cultured OCCM-30 control  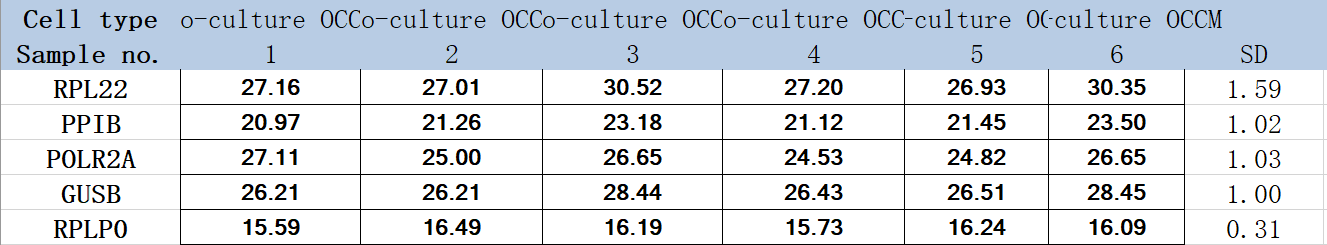 | Co-cultured OCCM-30 control  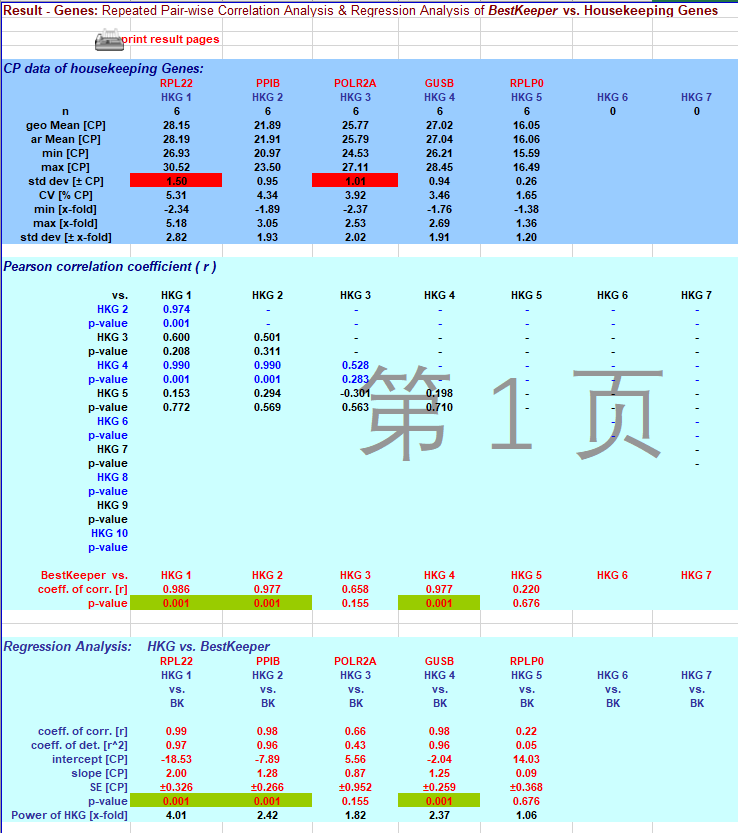 |
| 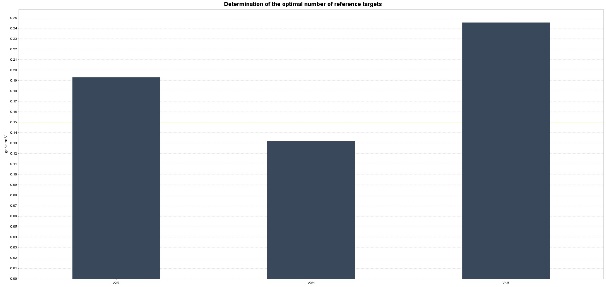  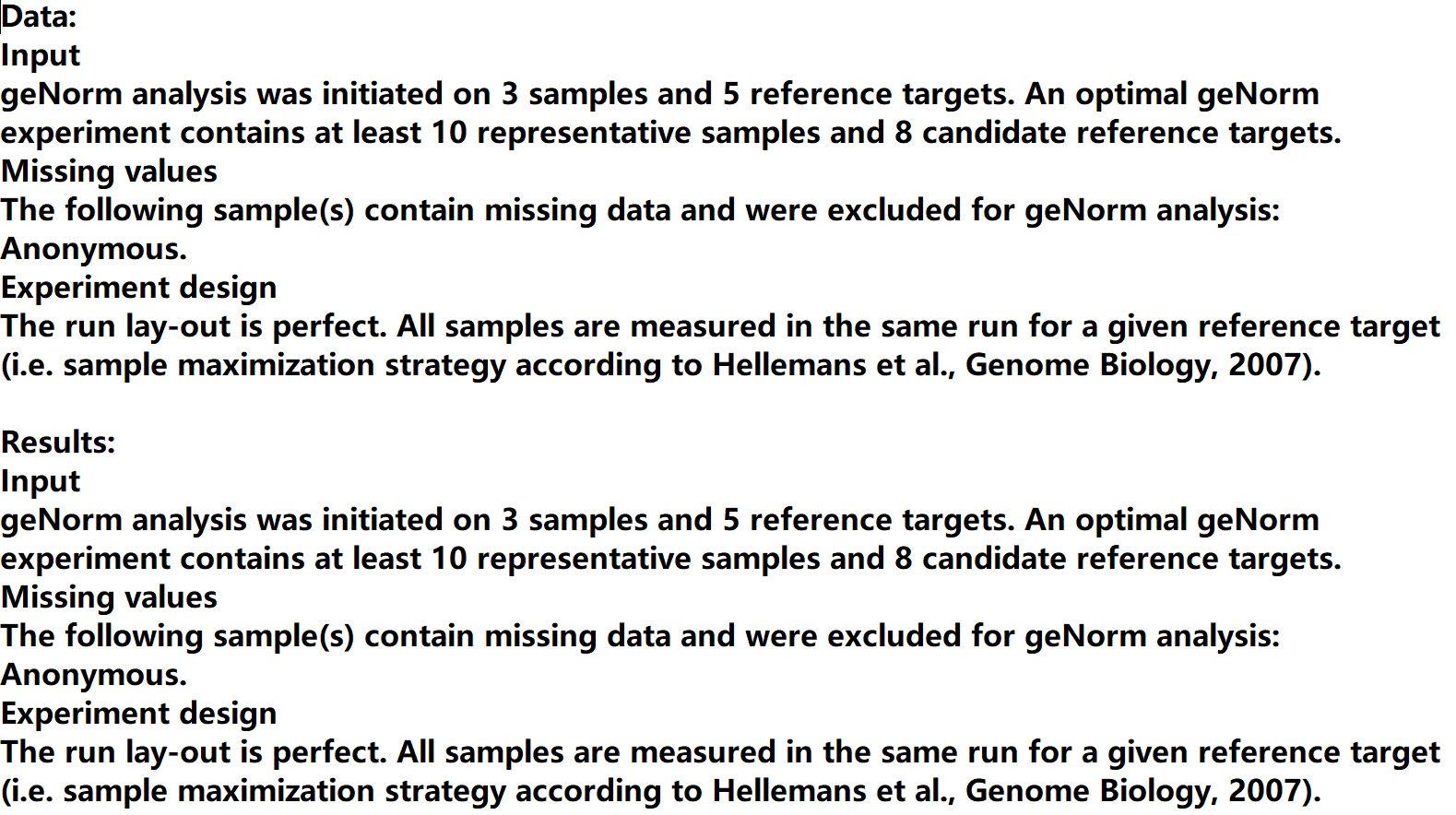 | Co-cultured OCCM-30 with 3 indicated time  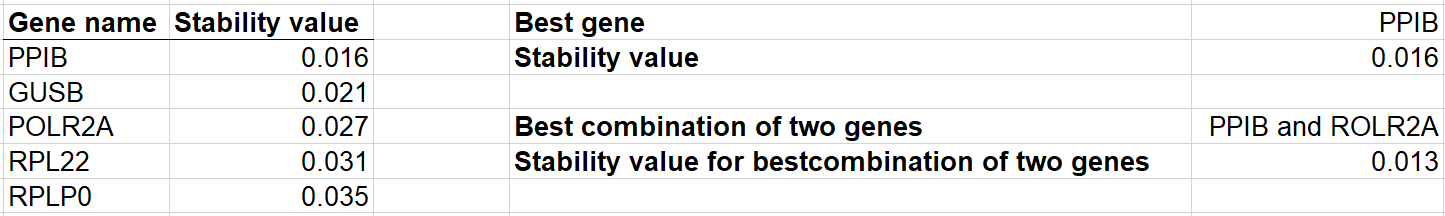 | Co-cultured OCCM-30 with 3 indicated time  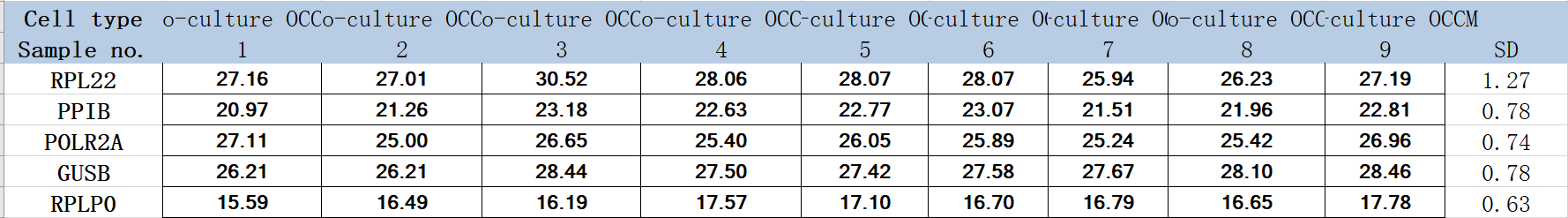 | Co-cultured OCCM-30 with 3 indicated time  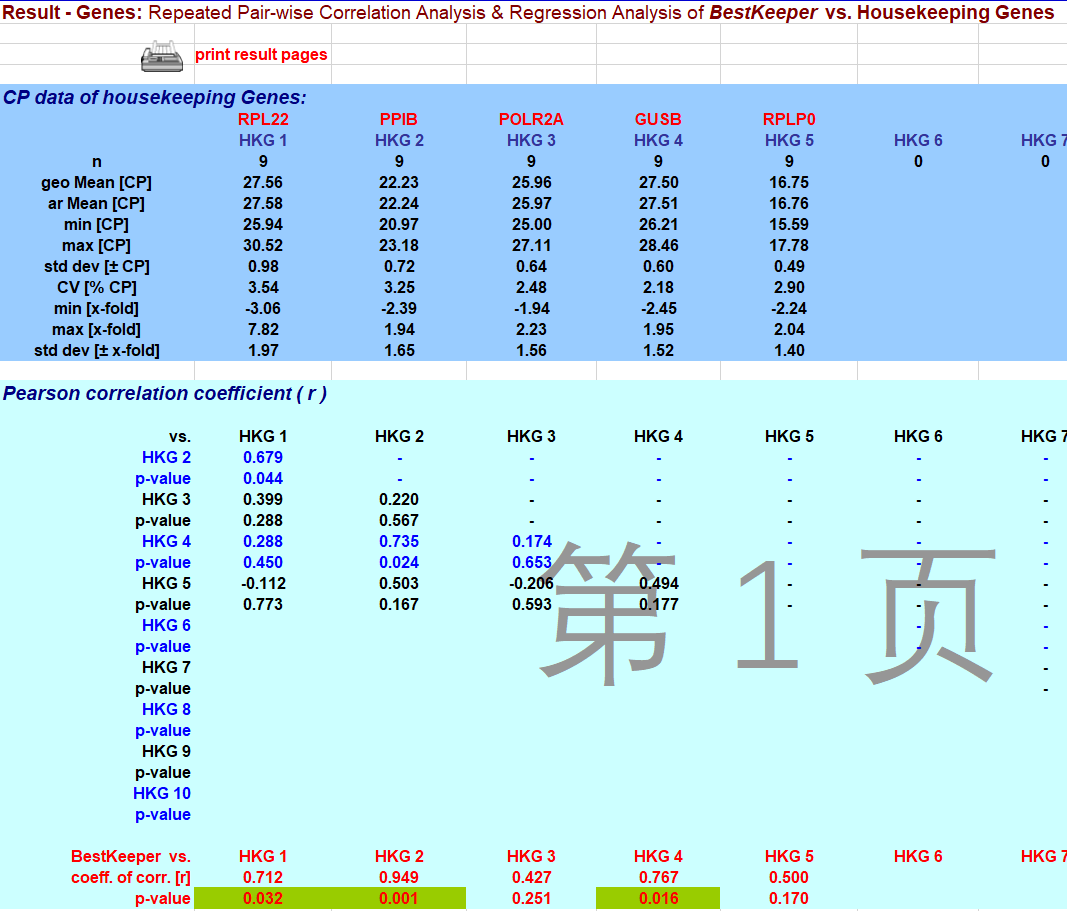 |
| ***Co-cultured SV-PDL control and with 3 indicated time*** | | | |
| geNorm | NormFinder | Comparative ΔCq | BestKeeper |
| 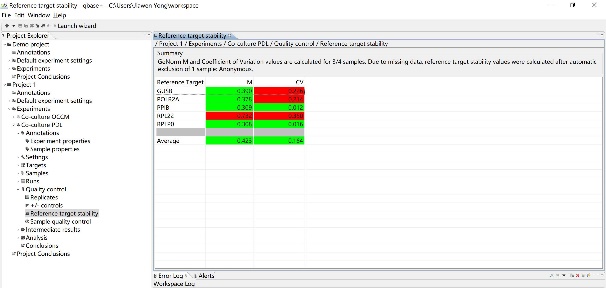  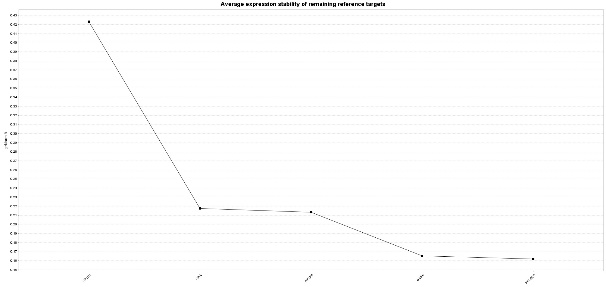 | Co-cultured SV-PDL control  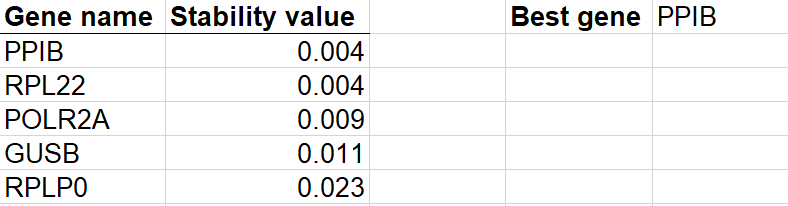 | Co-cultured SV-PDL control  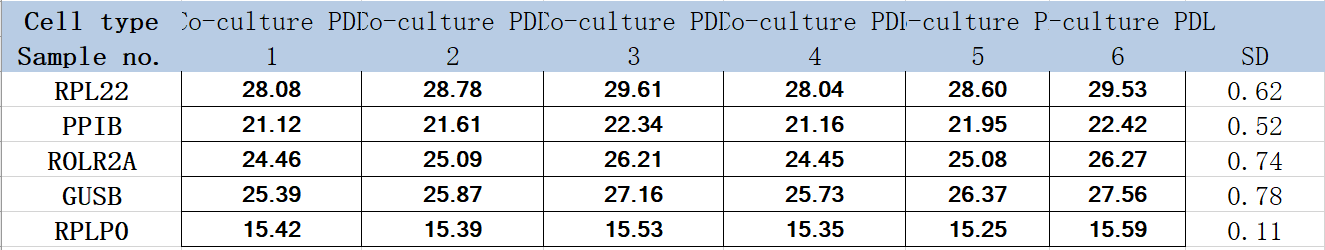 | Co-cultured SV-PDL control  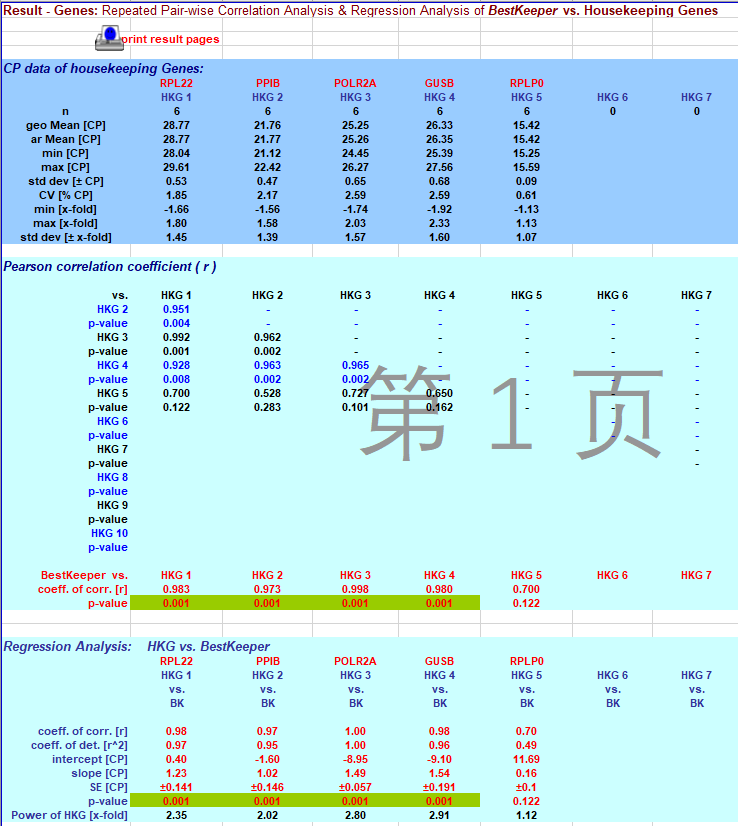 |
| 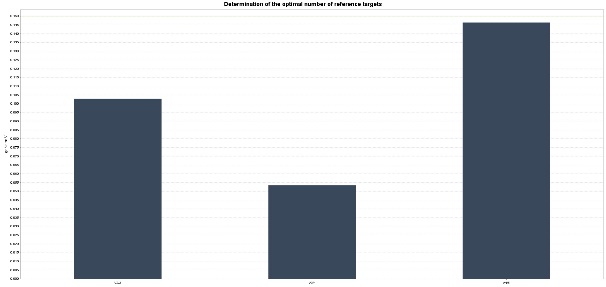  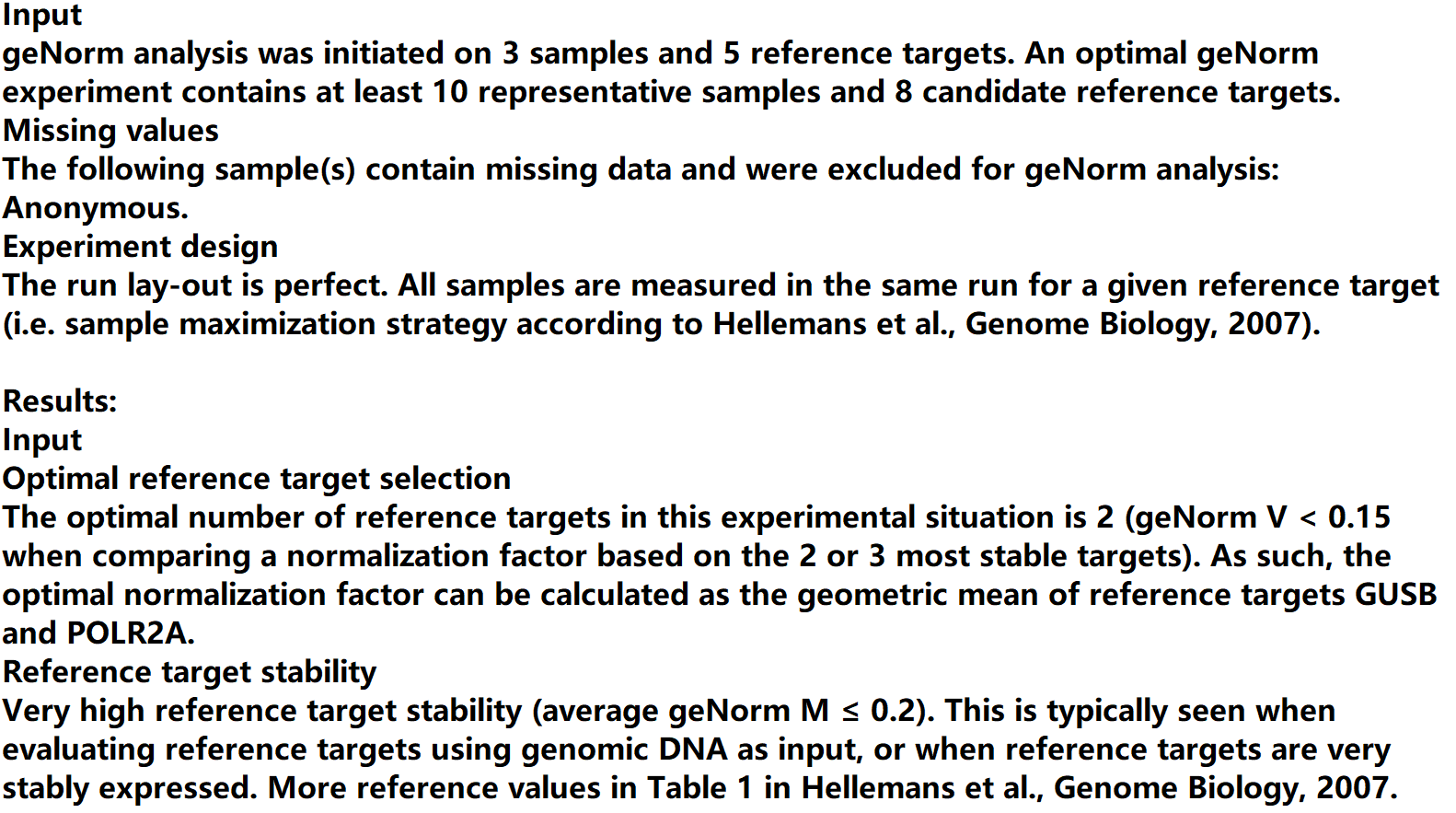 | Co-cultured SV-PDL with 3 indicated time  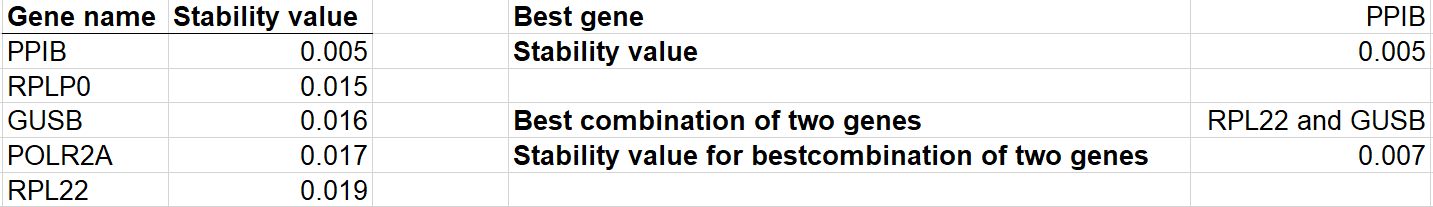 | Co-cultured SV-PDL with 3 indicated time  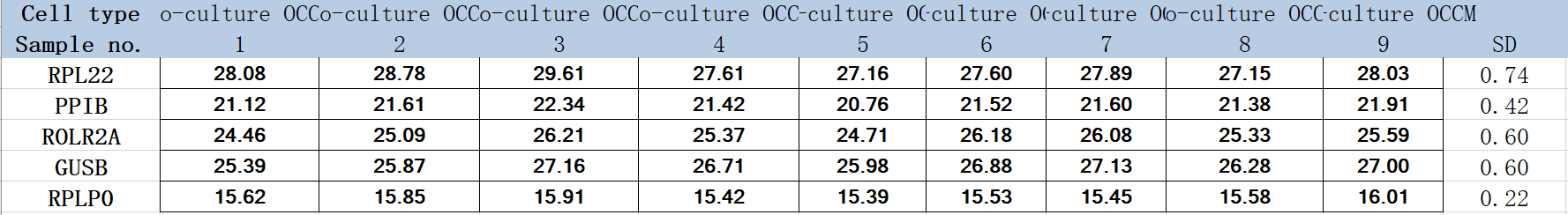 | Co-cultured SV-PDL with 3 indicated time  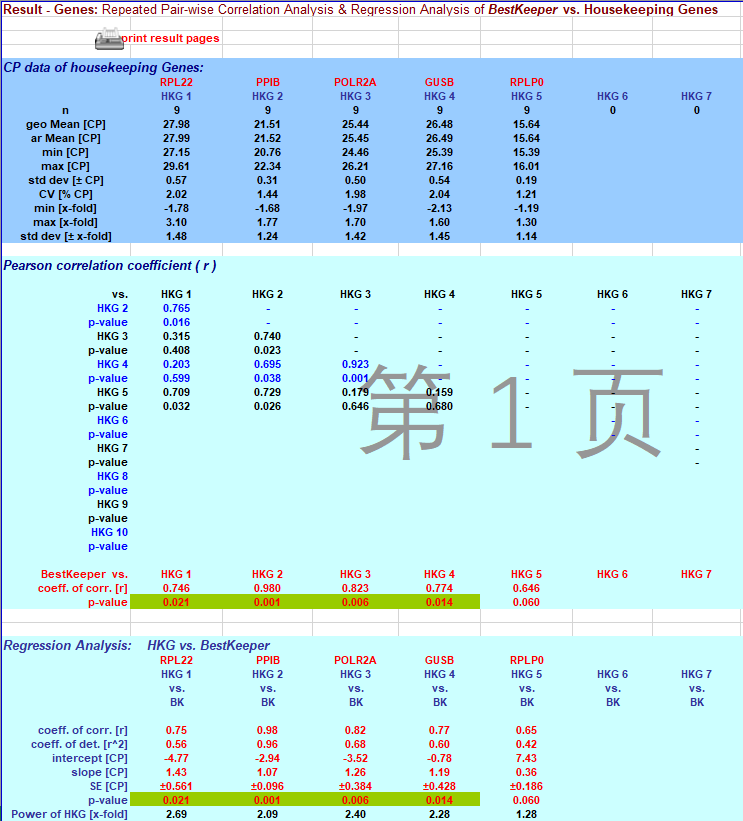 |

**Part 4. Original geNorm output data**

| ***Monocultured cementoblasts (OCCM-30)*** |
| --- |
| 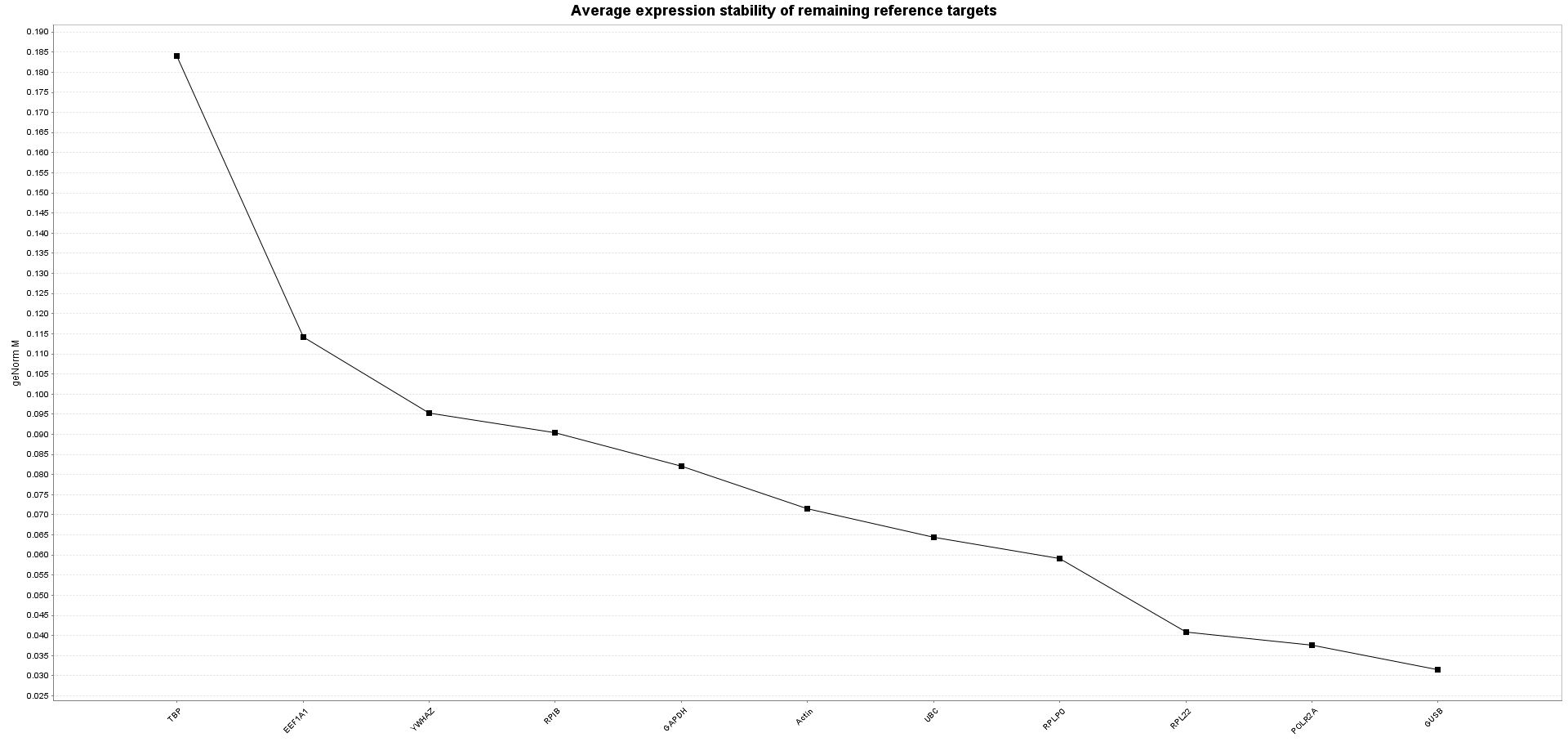 |
| 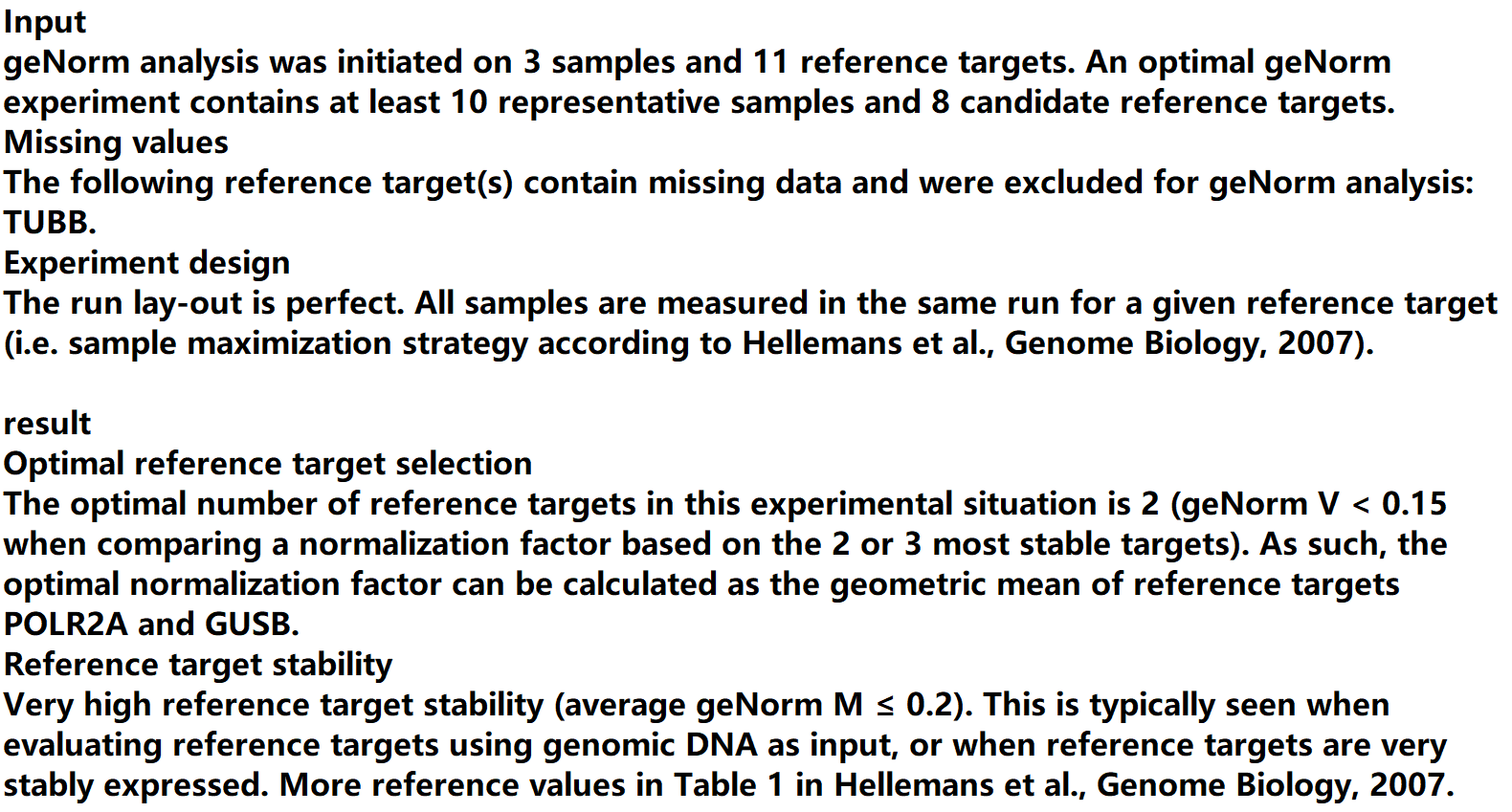 |

| ***Monocultured periodontal ligament cells (SV-PDL)*** |
| --- |
| ***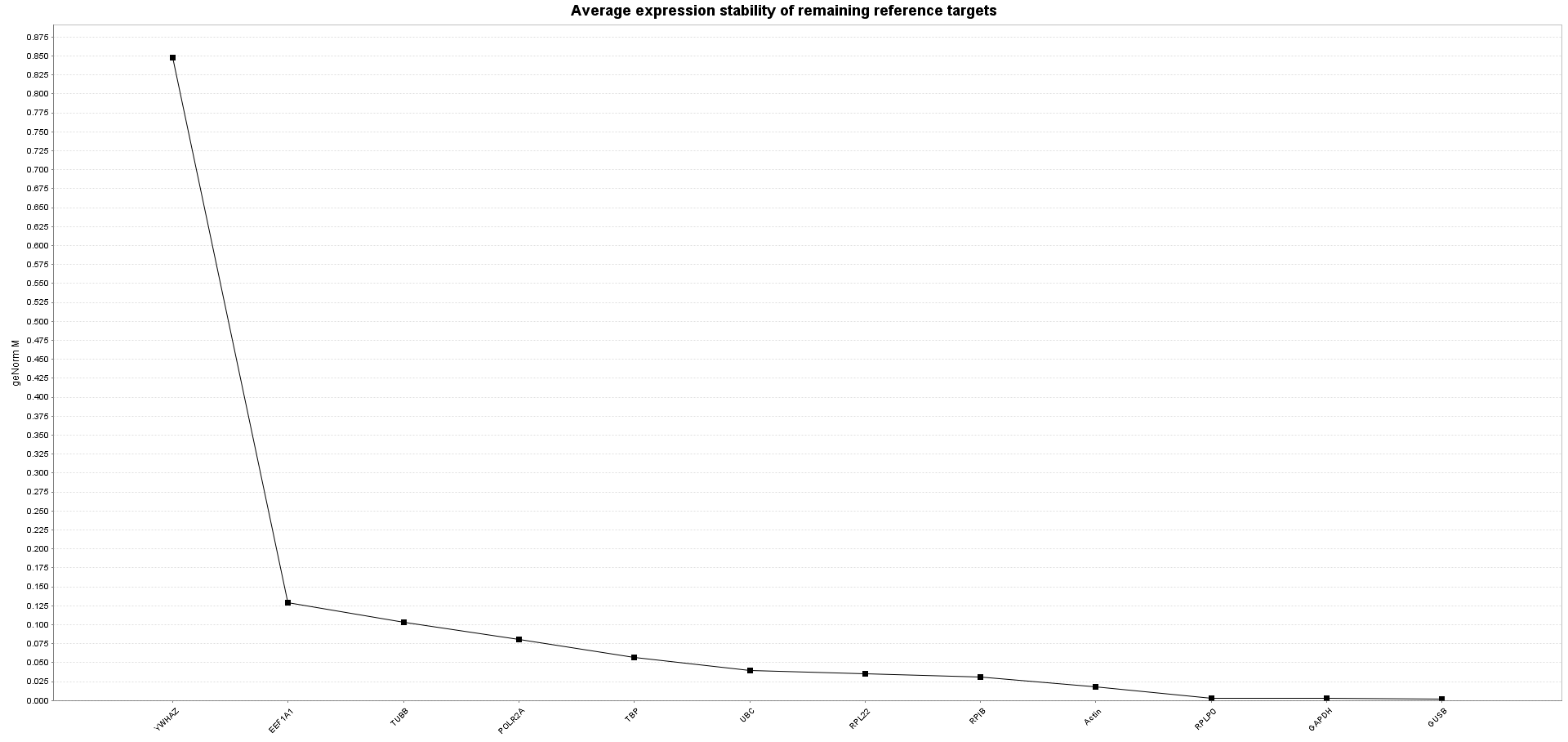*** |
| 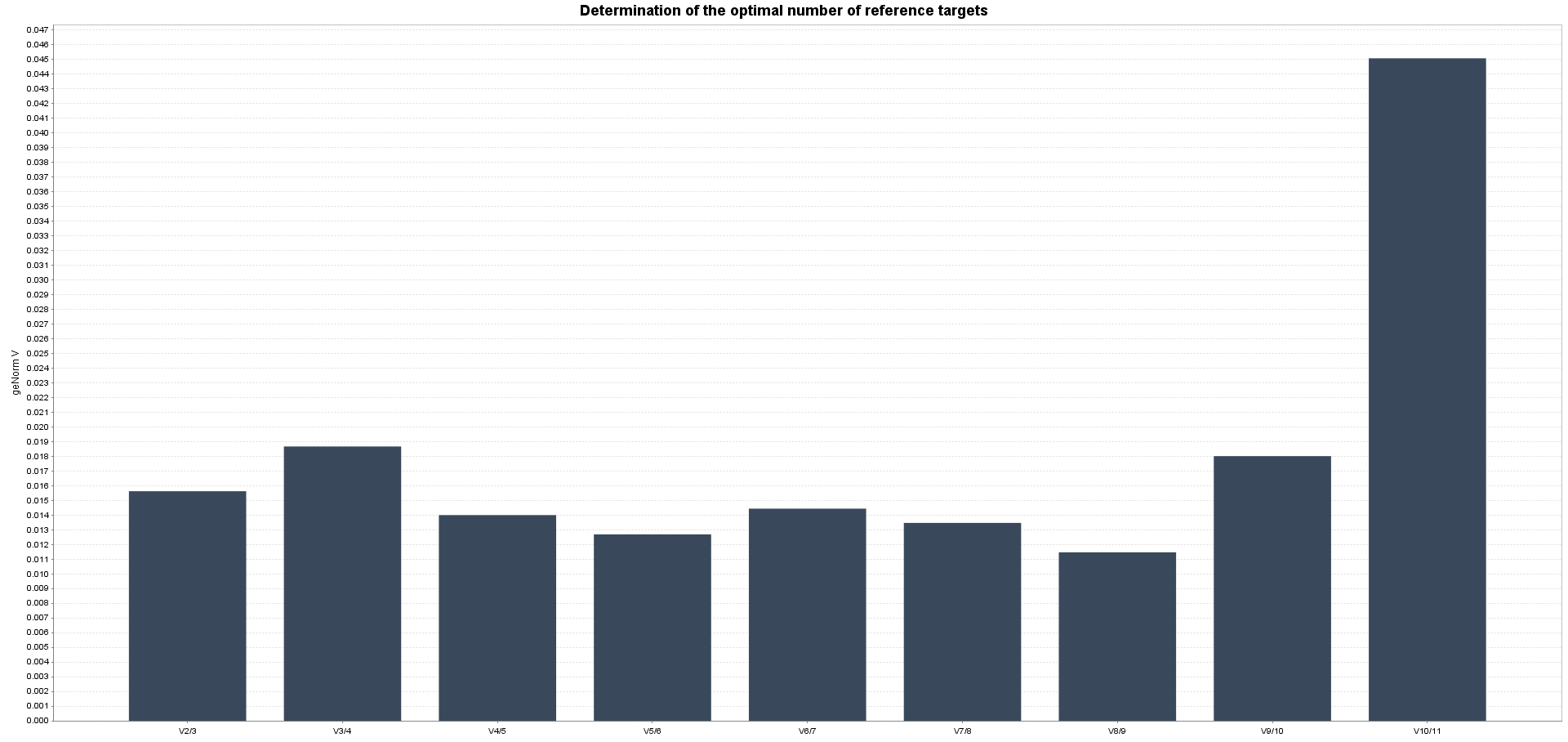 |
| 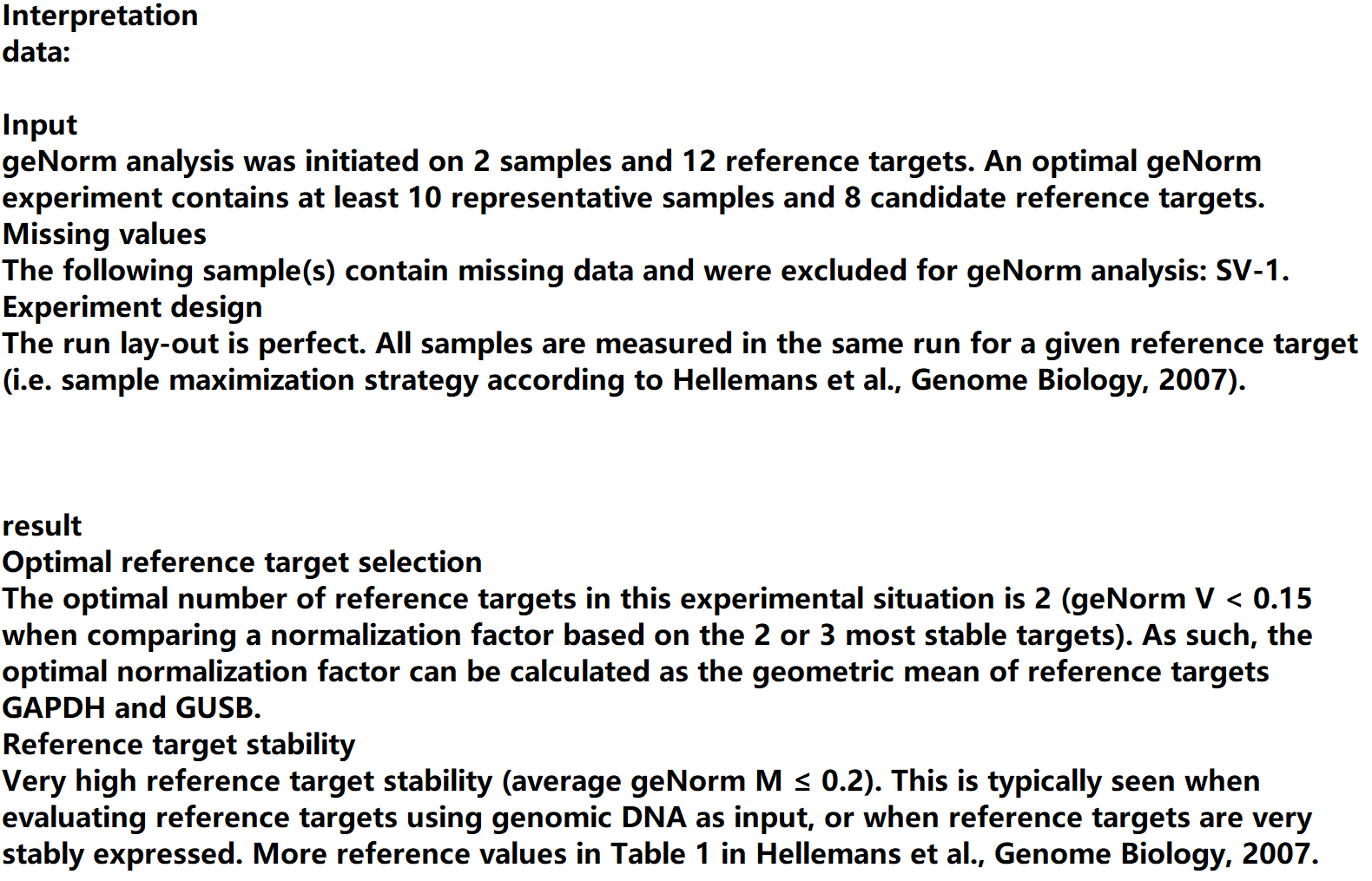 |
|  |
| ***Direct cell-cell contact cultured of OCCM-30 and SV-PDL*** |
| 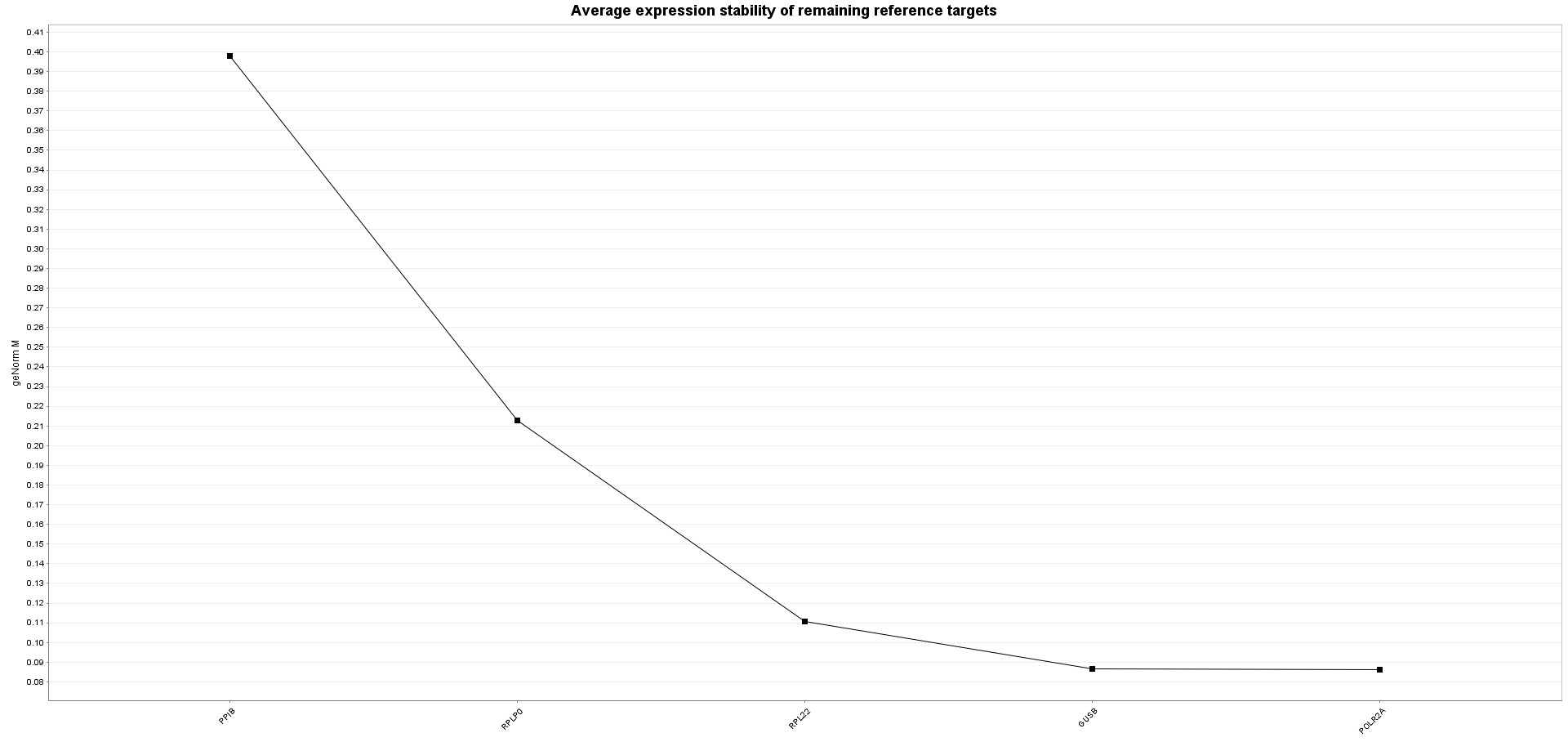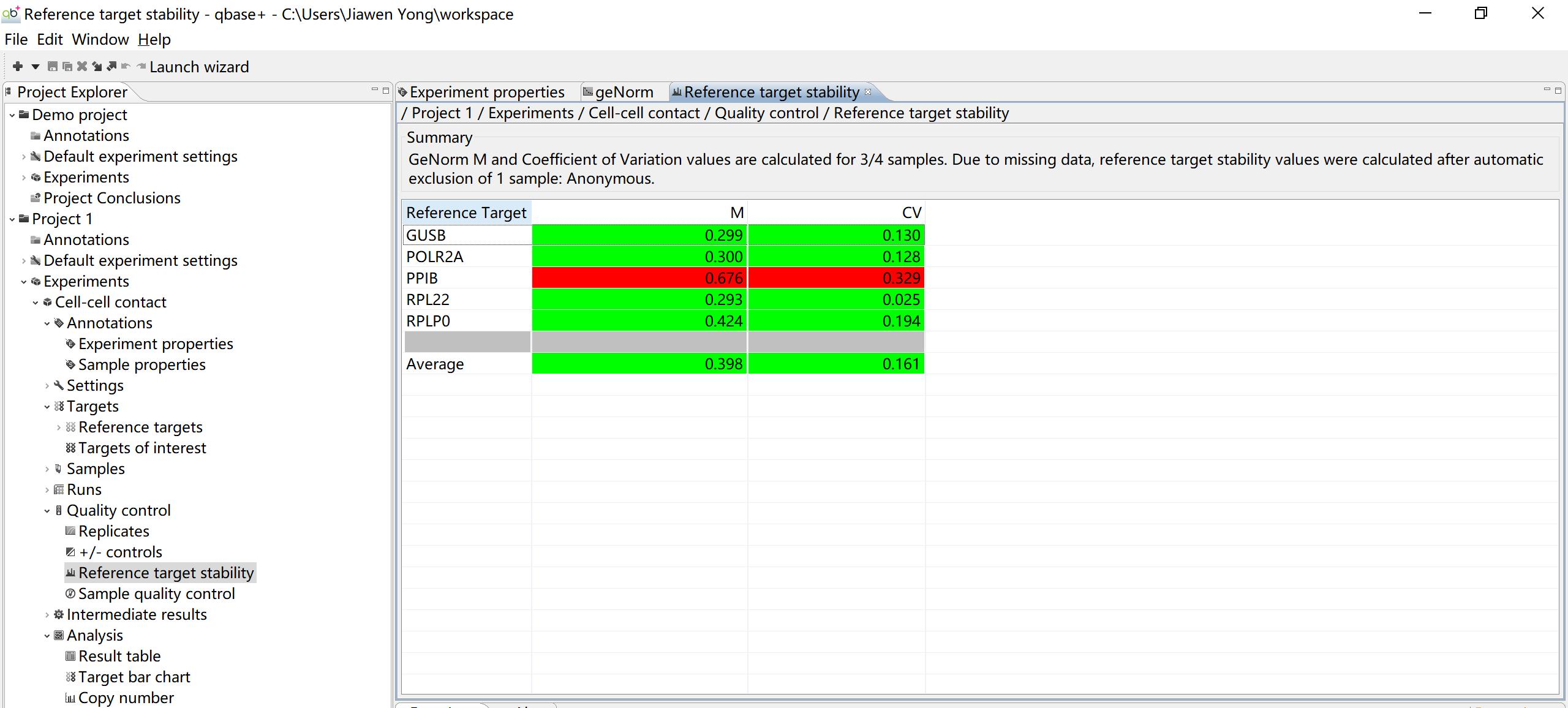 |
| 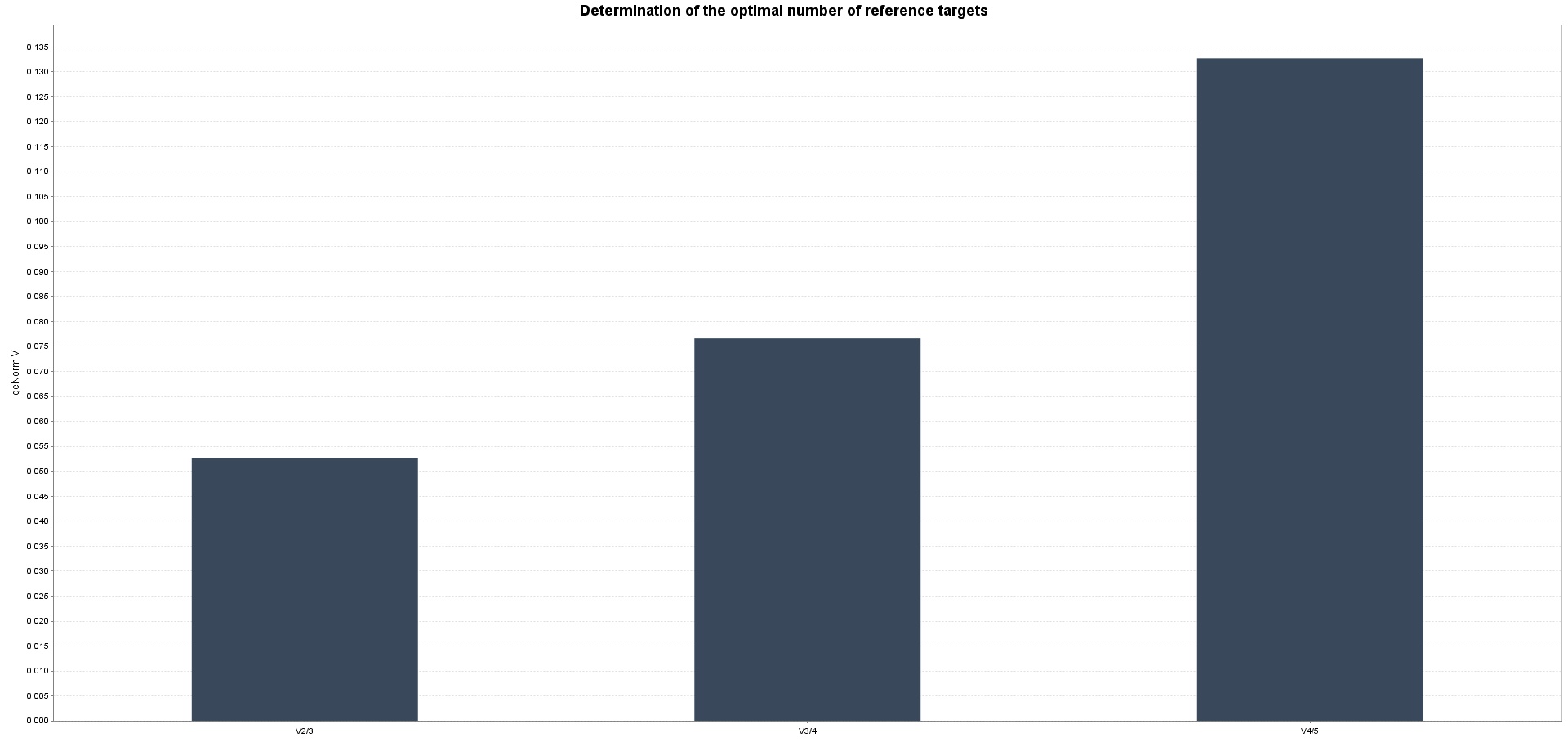 |
| 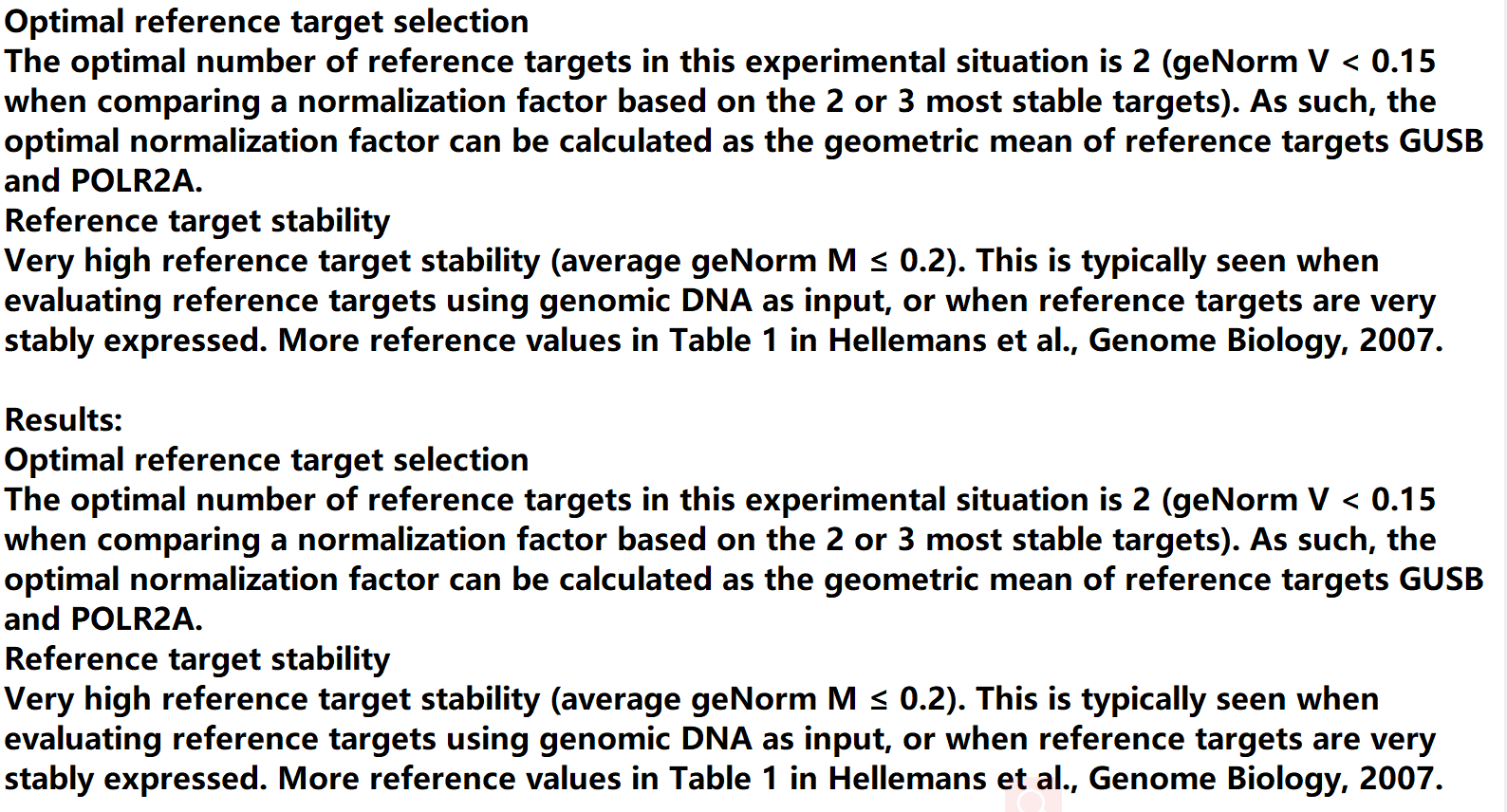 |

| ***Co-cultured OCCM-30 control and with 3 indicated time*** |
| --- |
| 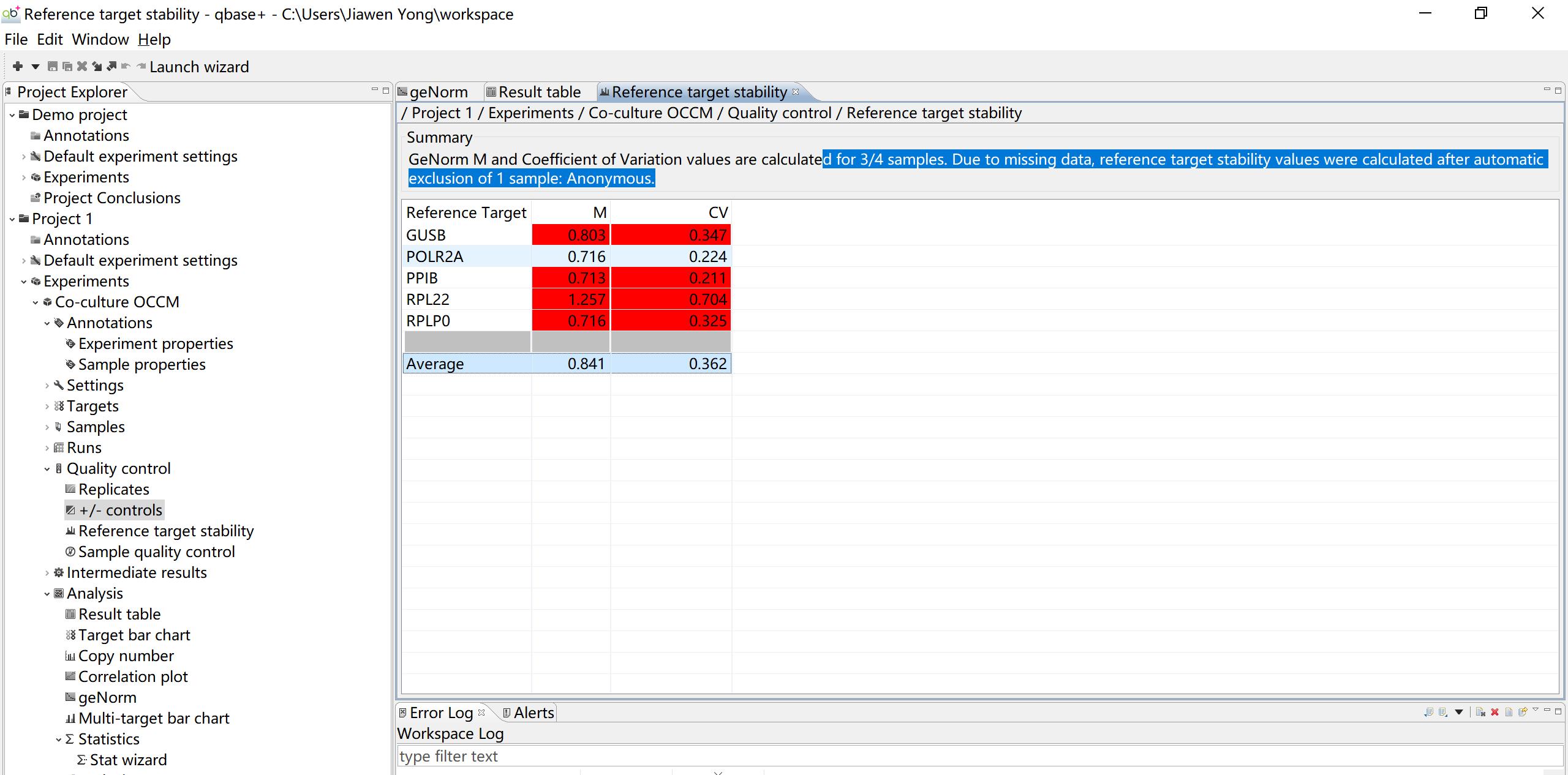 |
| 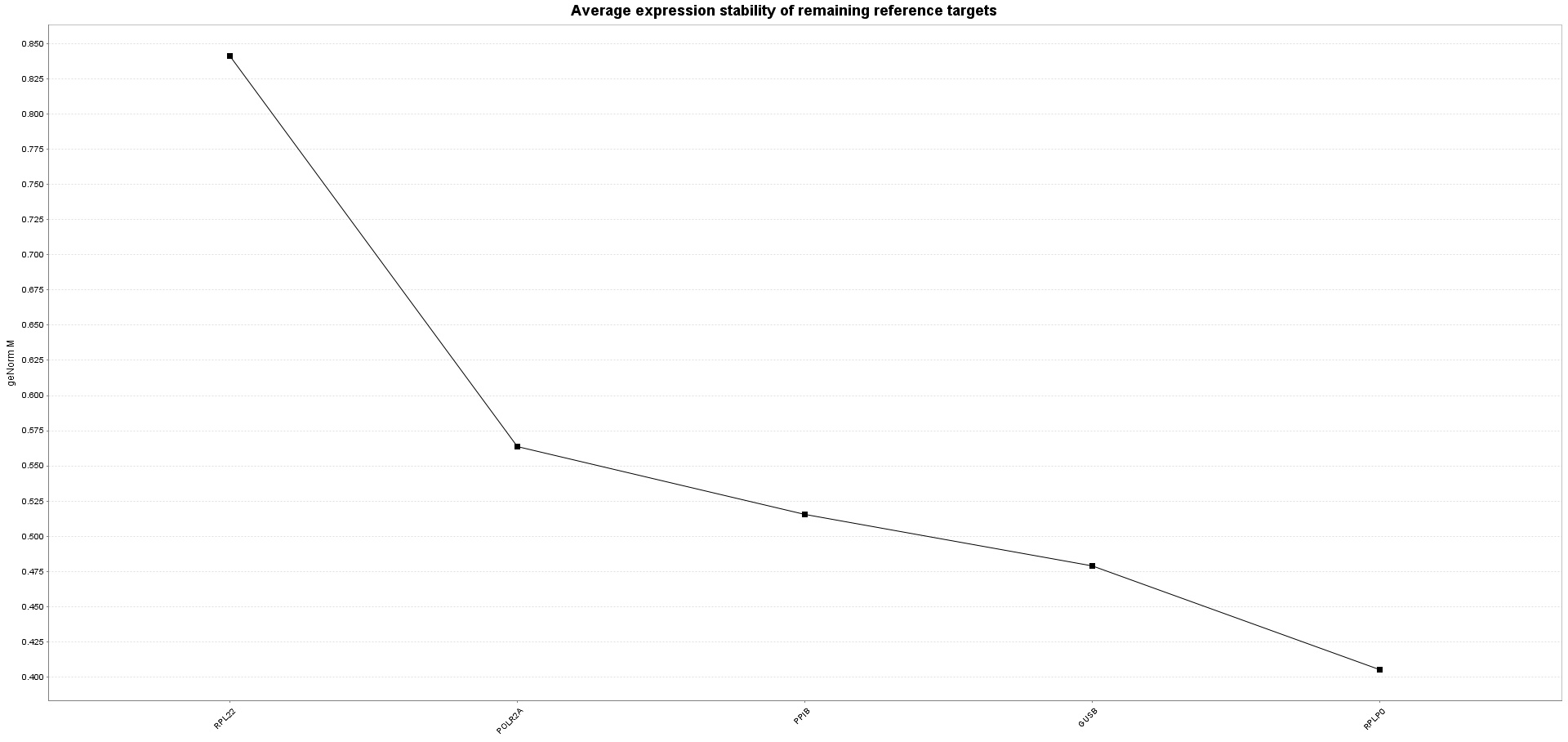 |
| 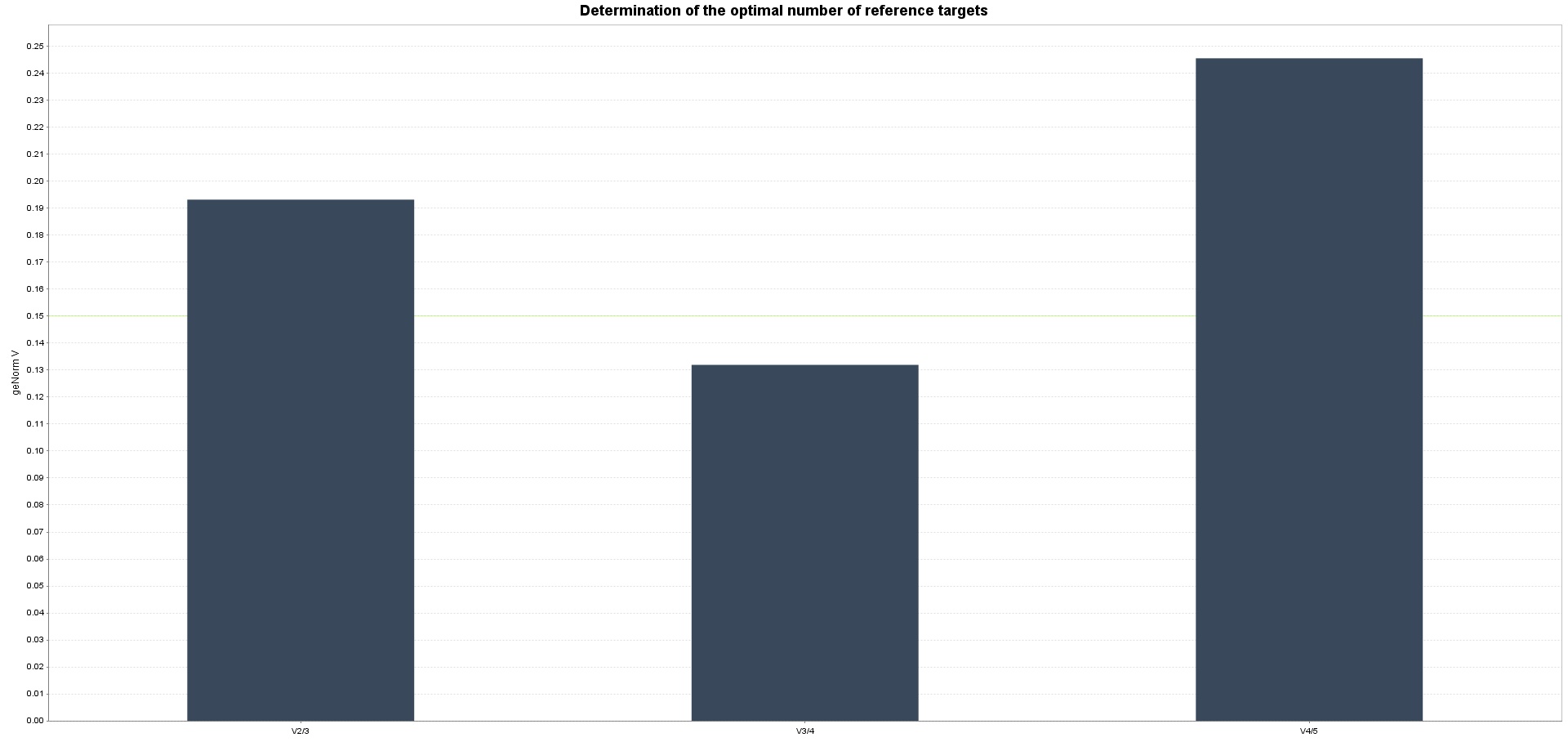 |
| 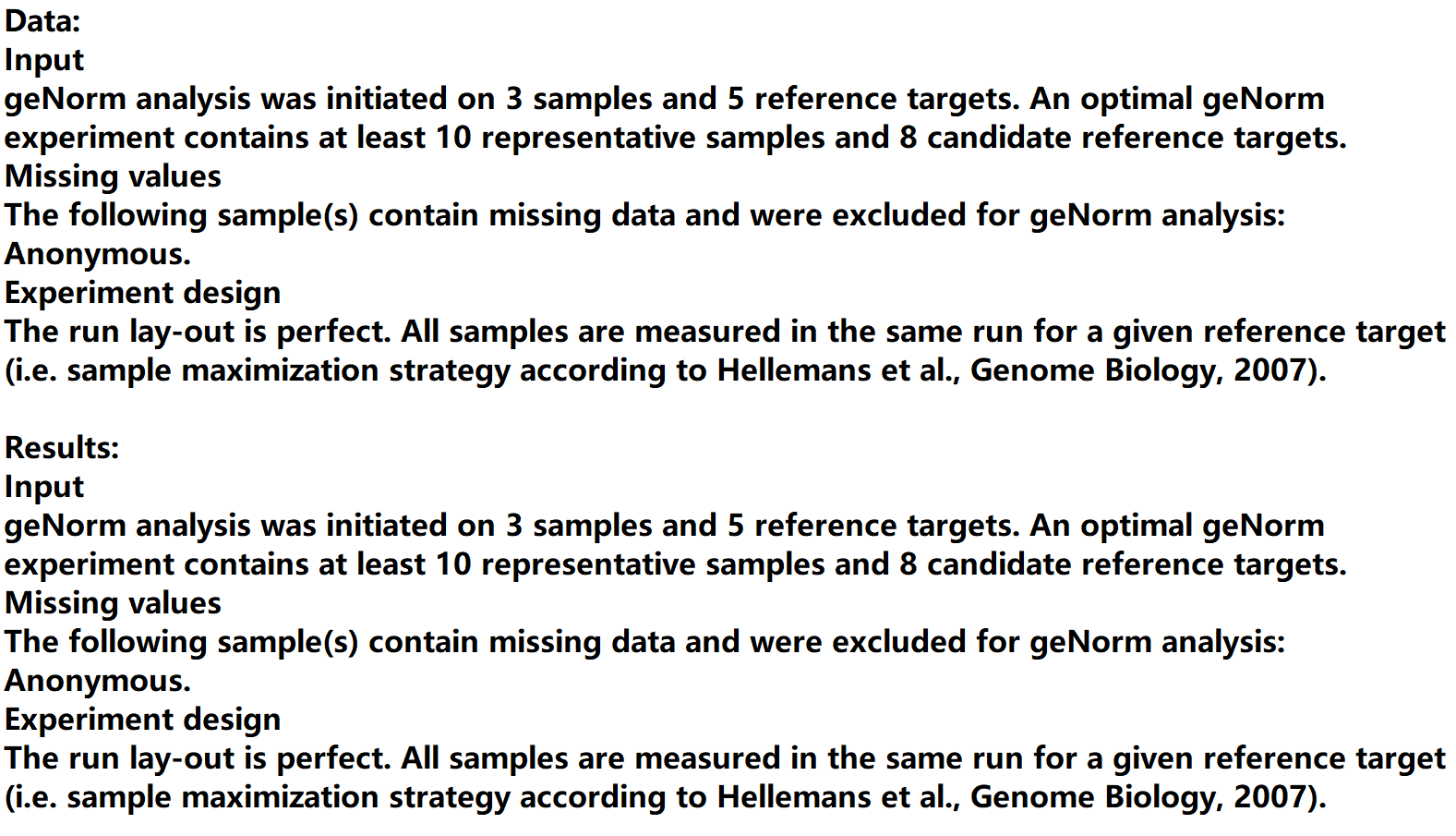 |

| ***Co-cultured SV-PDL control and with 3 indicated time*** |
| --- |
| 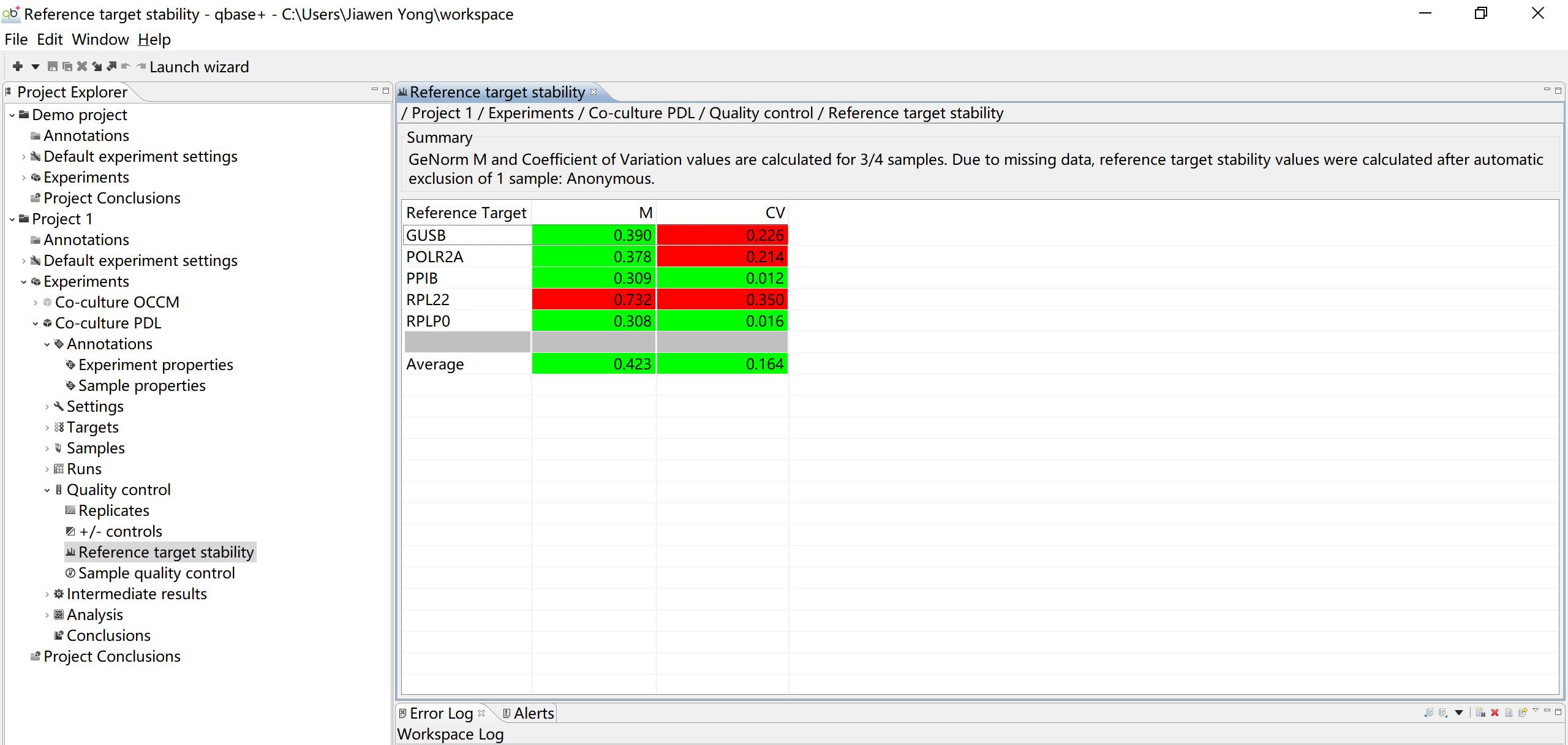 |
| 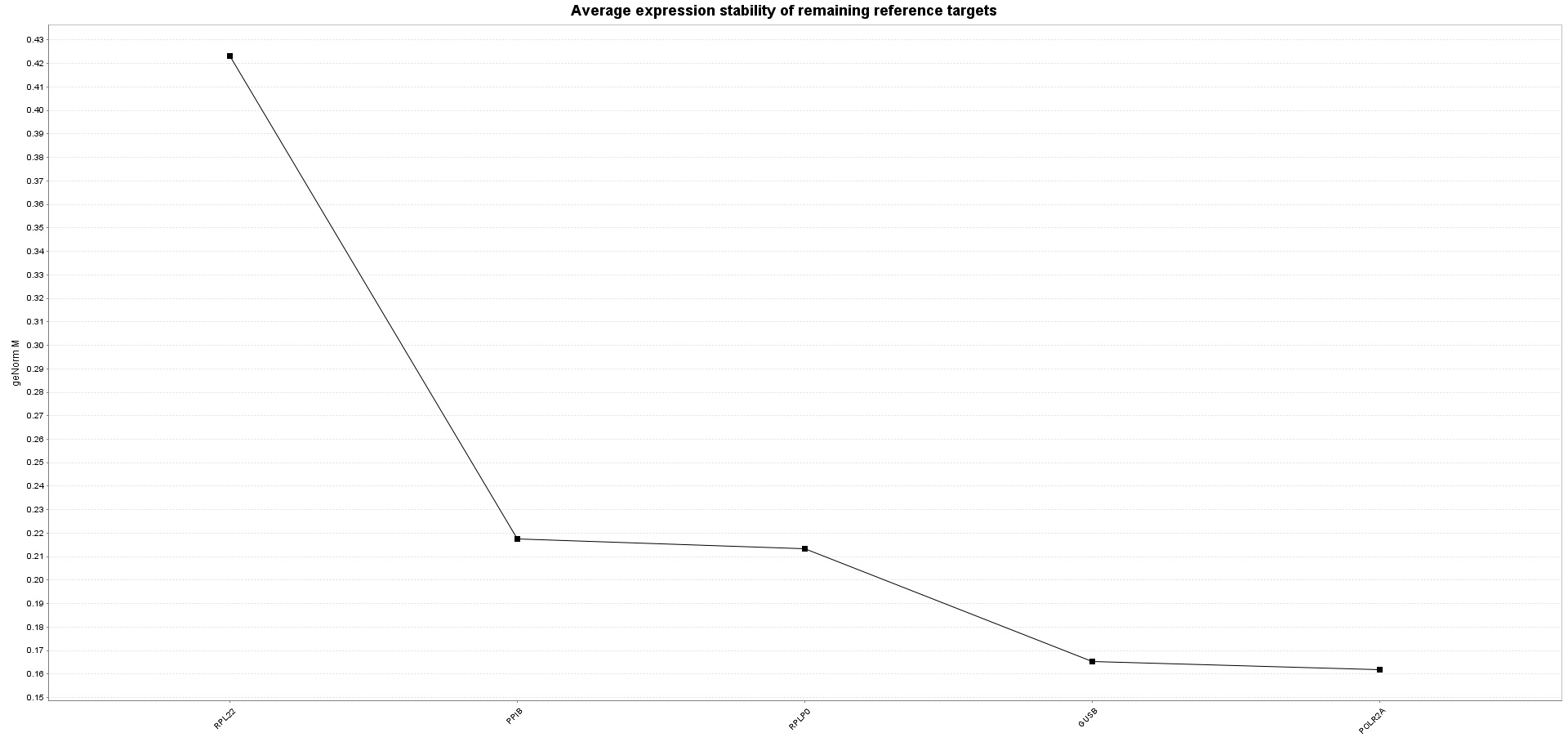 |
| 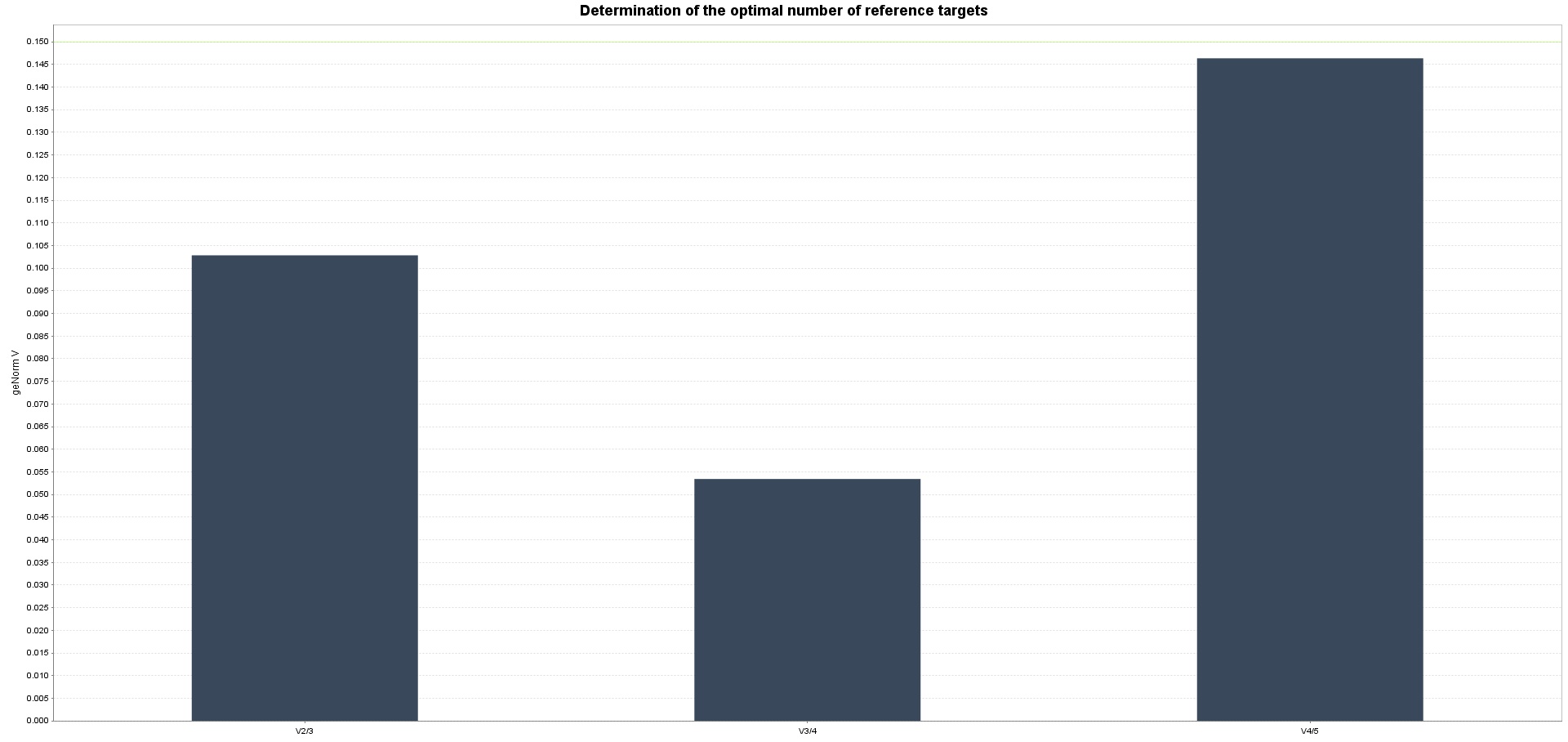 |
| 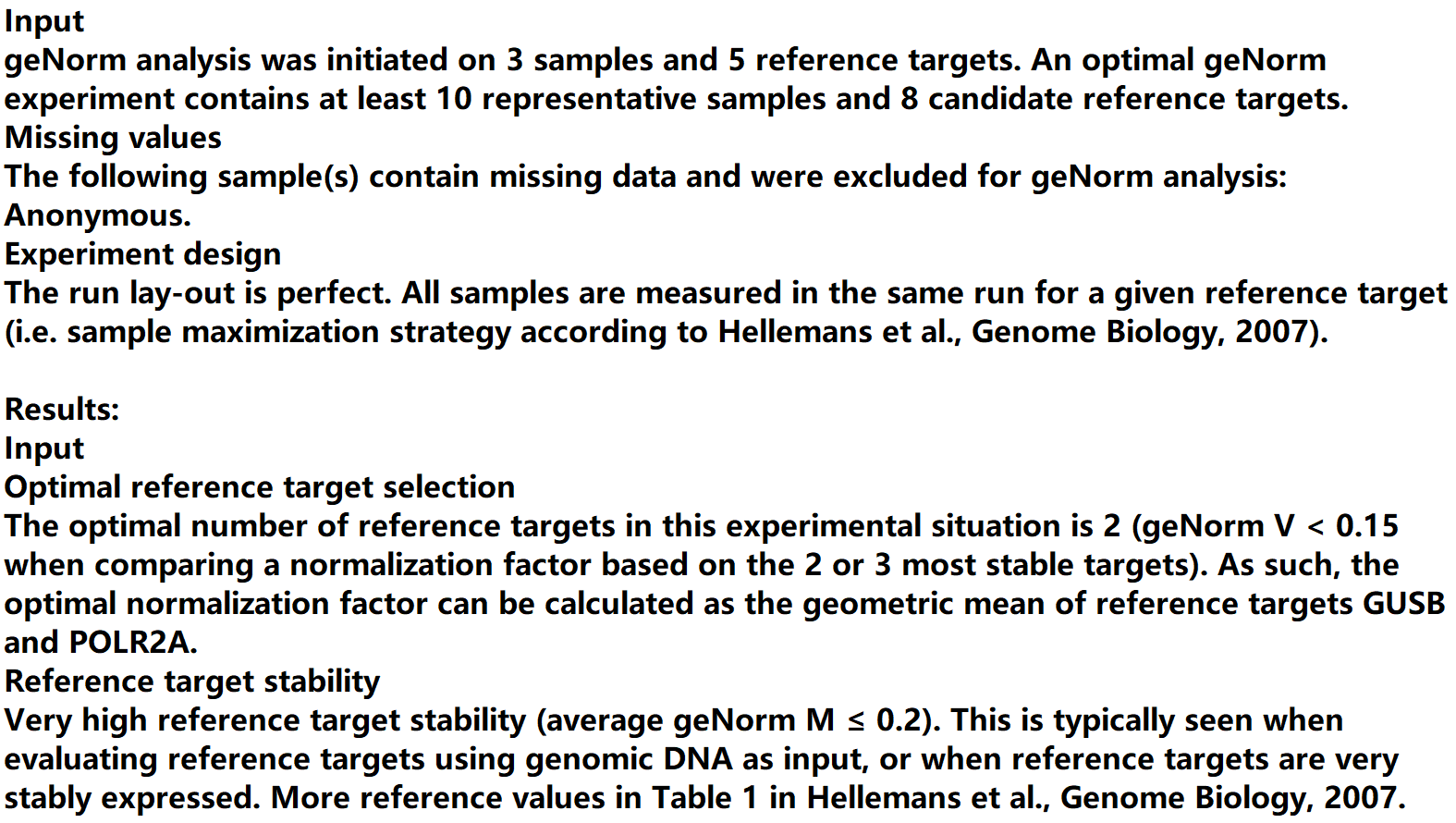 |

1. The upper limit of C_q_ cycles is set by 40, thus the qPCR system omits from the missing C_q_ values because the amplification cycle is above 40. [↑](#footnote-ref-1)
2. The missing C_q_ values in the dataset resulted from the technical problems. [↑](#footnote-ref-2)
3. The missing C_q_ values in the dataset resulted from the technical problems. [↑](#footnote-ref-3)
4. The missing C_q_ values in the dataset resulted from the technical problems. [↑](#footnote-ref-4)
